# Molecular, cellular and neurological consequences of infection by the neglected human pathogen Nocardia

**DOI:** 10.1186/s12915-022-01452-7

**Published:** 2022-11-09

**Authors:** Xingzhao Ji, Lichao Han, Weiying Zhang, Lina Sun, Shuai Xu, Xiaotong Qiu, Shihong Fan, Zhenjun Li

**Affiliations:** 1 Department of Pulmonary and Critical Care Medicine, Shandong Provincial Hospital Affiliated to Shandong First Medical University, Jinan, 250021 Shandong China; 2 Shandong Key Laboratory of Infections Respiratory Disease, Medical Science and Technology Innovation Center, Shandong First Medical University & Shandong Academy of Medical Sciences, Jinan, Shandong China; 3 State Key Laboratory for Infectious Disease Prevention and Control, National Institute for Communicable Disease Control and Prevention, Chinese Center for Disease Control and Prevention, 155 Changbai Road Changping District, Beijing, 102206 China; 4 Sericulture and Apiculture Research Institute, Yunnan Academy of Agricultural Science, Mengzi, 661100 Yunnan China

**Keywords:** *Nocardia farcinica*, Dual RNA-seq, Virulence factor, Parkinson’s disease, Microglia

## Abstract

**Background:**

*Nocardia* is a facultative intracellular pathogen that infects the lungs and brains of immunocompromised patients with consequences that can be fatal. The incidence of such infections is rising, immunocompetent individuals are also being infected, and there is a need to learn more about this neglected bacterial pathogen and the interaction with its human host.

**Results:**

We have applied dual RNA-seq to assess the global transcriptome changes that occur simultaneously in *Nocardia farcinica* (*N. farcinica*) and infected human epithelial alveolar host cells, and have tested a series of mutants in this in vitro system to identify candidate determinants of virulence. Using a mouse model, we revealed the profiles of inflammation-related factors in the lung after intranasal infection and confirmed that nbtB and nbtS are key virulence genes for *Nocardia* infection in vivo. Regarding the host response to infection, we found that the expression of many histones was dysregulated during the infection of lung cells, indicating that epigenetic modification might play a crucial role in the host during *Nocardia* infection. In our mouse model, *Nocardia* infection led to neurological symptoms and we found that 15 of 22 *Nocardia* clinical strains tested could cause obvious PD-like symptoms. Further experiments indicated that *Nocardia* infection could activate microglia and drive M1 microglial polarization, promote iNOS and CXCL-10 production, and cause neuroinflammation in the substantia nigra, all of which may be involved in causing PD-like symptoms. Importantly, the deletion of nbtS in *N. farcinica* completely attenuated the neurological symptoms.

**Conclusions:**

Our data contribute to an in-depth understanding of the characteristics of both the host and *Nocardia* during infection and provide valuable clues for future studies of this neglected human pathogen, especially those addressing the underlying causes of infection-related neurological symptoms.

**Supplementary Information:**

The online version contains supplementary material available at 10.1186/s12915-022-01452-7.

## Background

Nocardiosis is an infectious disease caused by the ubiquitous, intracellular gram-positive aerobic bacterium *Nocardia spp*. Nocardiosis has been treated as an “opportunistic” disease that is mainly involved in immunocompromised patients. However, an increasing number of nocardiosis cases in immunocompromised individuals are being reported [1]. It has been reported that approximately 500 to 1000 cases of nocardiosis infections occur every year in the USA [2]. In recent years, with the increasing number of HIV patients, organ transplant patients, and the aging population, the infection rate of *Nocardia* has gradually increased. *Nocardia* predominantly causes infection in the lung and brain, and it can disseminate via the blood to cause infection in almost all organs. It will be life threatening when it disseminates to the central nervous system (CNS), with mortality rates as high as 85% in immunocompromised individuals [2]. Immunocompetent nocardia patients will have to get antibiotic treatment for 6 to 12 months and immunocompromised patients or those with CNS dissemination should receive treatment for at least 12 months [3]. At present, trimethoprim–sulfamethoxazole is the preferred therapy for nocardial infections [4]. Due to the nonspecific symptoms of infection, the long culture period, and the lack of specific diagnostic reagents, *Nocardia* has been underrecognized, underdiagnosed, and neglected [5]. If it cannot be diagnosed and treated in time, especially for patients with immunodeficiency, it will be fatal.

Current research on *Nocardia* is limited. *Nocardia* can invade and survive in host cells, such as epithelial cells and macrophages, and can resist the host immune response by producing a variety of virulence factors, such as superoxide and hemolysin [6, 7]. Previously, we reported that the *mce*, *hbha*, and *nfa34810* genes are involved in adhesion and invasion as virulence factors in *Nocardia* [8, 9]. Data from the complete genome sequence showed that the genome of *N. farcinica* contains several putative virulence genes, such as *catalases* and *nbt*, which may play a crucial role during the infection process [10]. Unfortunately, the role of most of the above genes in *Nocardia* during infection has not yet been investigated.

*Nocardia* can quickly traverse capillary endothelial cells to enter the brain parenchyma and cause brain infections. Richter et al. reported for the first time that a patient infected with *Nocardia* had neurological symptoms at 6 weeks post-infection, such as mask face, trembling movement, stiffened muscles, and irregular limb tremors [11]. They found that *Nocardia* preferentially invaded the substantia nigra and putamen without causing apparent inflammation in both mice and *Macaca fasicularius*, and further study showed that head shake symptoms could be stopped temporarily after treatment with L-dopa [12]. David et al. documented that the neurological symptoms caused by *Nocardia* infection may be related to causes such as inner ear pathology; however, whether *Nocardia* infection causes PD remains to be clarified [13]. At present, research on inflammation of the nervous system caused by *Nocardia* infection is scarce, and the mechanism of *Nocardia* infection causing neurological symptoms needs to be examined in the future.

Dual RNA-seq can simultaneously analyze the transcriptional profile in both hosts and pathogens during infection. Rieza et al. used dual RNA-seq to simultaneously study the interaction of pneumococcus and lung epithelium and revealed novel cellular processes and metabolic rewiring during pneumococcal infection [14]. Buket et al. analyzed infection-linked transcriptome adaptation in *Haemophilus influenzae* and host cells and revealed regulatory responses and metabolism modulation via dual RNA-seq, thus providing key insights into *Haemophilus influenzae* pathogenesis and the development of prevention strategies [15]. However, RNA-seq data for *Nocardia*, as emerging or neglected pathogens, are lacking, which merits the application of transcriptome data through dual RNA-seq technology to fill the gaps in *Nocardia*-related research fields.

Taking advantage of dual RNA-seq advances, we first describe the transcriptional profile in both *Nocardia* and host cells. We combined the immune profiles of host cells from sequencing data with an in vivo assay to describe the characteristics of cytokine secretion in hosts post-infection with *Nocardia*. A series of novel virulence factors from *Nocardia* were found, and further in vivo experiments confirmed that iron acquisition genes played a key role in *Nocardia* infection. For host cells, we found that epigenetic modification might play an important role in the response to *Nocardia* infection. In vivo, we confirmed the neurological symptoms of mice post-infection with *N. farcinica* and analyzed 22 clinical strains that may cause neurological symptoms in mice. Mechanistically, the M1 microglial polarization induced by *N. farcinica* might be involved in neuroinflammation, which was related to the loss of dopaminergic neurons in the substantia nigra and decreased dopamine content in the striatum. Furthermore, the deletion of *nbtS* in *N. farcinica* completely attenuated the neurological symptoms. Our data present the first transcriptome analysis of *Nocardia* during interaction with alveolar epithelial cells and provide novel insights into *Nocardia* pathogenesis. More importantly, our study will effectively fill the gap in the research field of *Nocardia* and provide a theoretical basis for further in-depth system understanding and research on emerging or neglected pathogens.

## Results

### Dual RNA-seq analysis of epithelial cells and Nocardia during infection

**Fig. 1:**
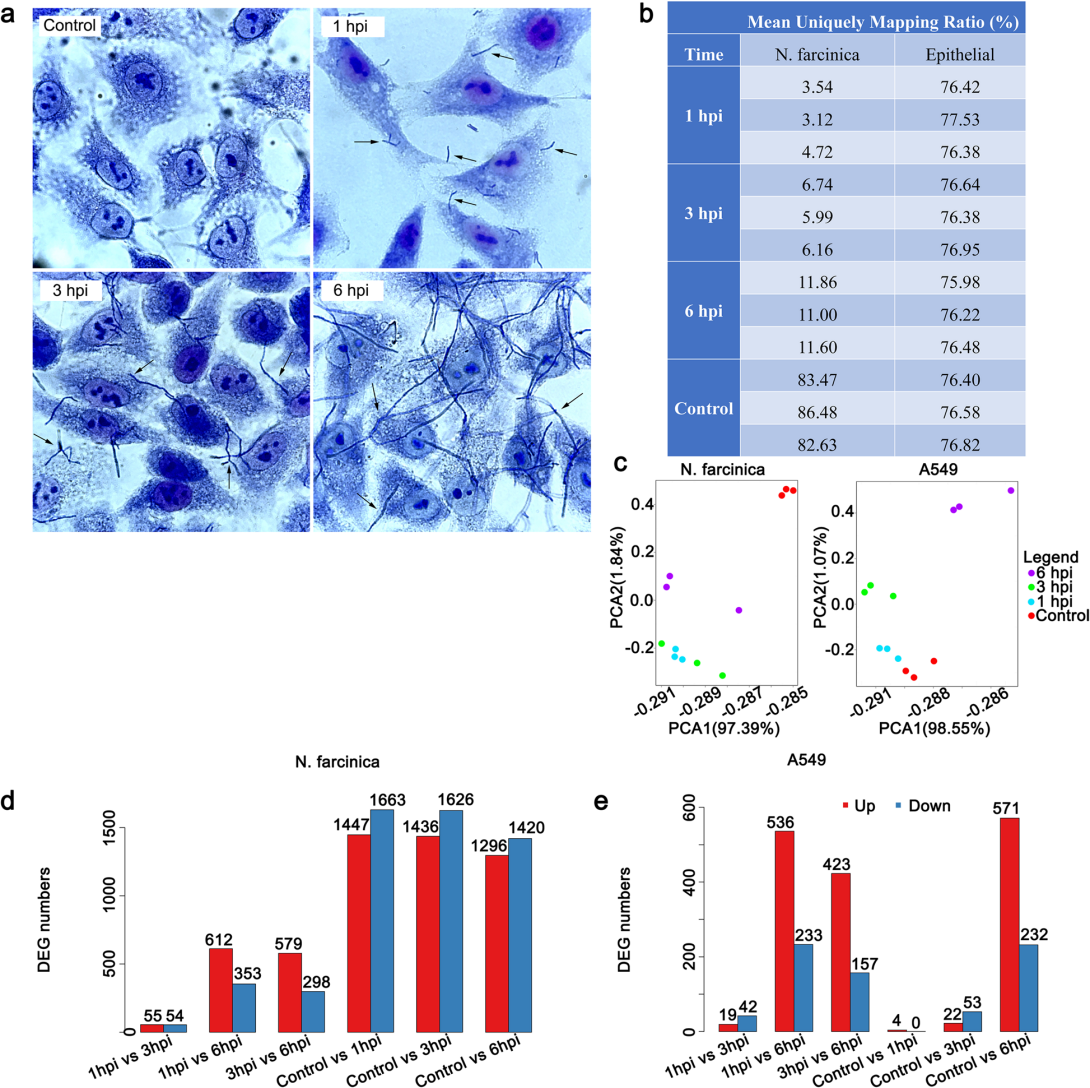
Overview of the dual RNA-seq data. **a** Giemsa images of A549 cells infected with *N. farcinica* at different time points post-infection. **b** Sequencing reads containing low-quality, adaptor-polluted, and a high content of unknown base reads were removed and mapped to the reference genome. **c** PCA. Points represent each sample. The samples in one group are the same color. **d**, **e** Summary of DEGs of *N. farcinica* (**d**) and A549 cells (**e**) during infection

To date, no RNA-seq data related to *Nocardia* infection have been reported. To fully understand the pathogenic mechanism of *Nocardia* and the adaptive response mechanism of the host response to *Nocardia* infection, the dual RNA-seq approach was applied in this study. To characterize the interactions of *N. farcinica* with lung epithelial cells, an in vitro infection model was built using human alveolar epithelial cells. *N. farcinica* was used to infect A549 cells for 1, 3, and 6 h at an MOI of 10. Giemsa analysis showed that some *N. farcinica* adhered and invaded the cells (Fig. 1a).

Dual RNA-seq was used to determine genome-scale expression events in both hosts and bacteria at 1, 3, and 6 h post-infection (hpi). The transcriptomic data for dual RNA-seq were generated by applying the paired-end 75-nucleotide sequence method. Approximately 120 to 200 million reads were obtained at each time point after depletion of rRNA in both cells and bacteria, which was sufficient for dual RNA profiling (Fig. 1b). Principal component analysis (PCA) was used to investigate the trends in sequence data, which showed that similar samples clustered together without obvious batch effects (Fig. 1c). Pearson correlation of between samples is shown in Additional file 1: Fig. S1.

To simplify further analyses, we selected genes that were differentially expressed ≥2-fold with an adjusted *p*<0.05 for follow-up research (Fig. 1d, e). Kyoto Encyclopedia of Genes and Genomes (KEGG) pathway analysis indicated that these differentially expressed genes (DEGs) were mainly involved in membrane transport, translation, amino acid, and carbohydrate metabolism for *N. farcinica*, transport and catabolism, cell growth and death, signal transduction, signaling molecules and interaction, immune diseases, substance dependence, metabolism, and immune system for host cells (Additional file 1: Fig. S2 and Fig. S3).

To validate the dual RNA-seq data, we first applied the total RNA that was used for sequencing as a template and randomly selected DEGs at different time points of infection for verification. Then, we repeated the experiments to obtain total RNA as a template and randomly selected DEGs for verification (Additional file 1: Table S1). As shown in Additional file 1: Fig. S4, the RNA-seq and qRT–PCR data showed a relatively strong correlation: *R*^*2*^ = 0.95 for *Nocardia* and *R*^*2*^ = 0.86 for epithelial cells, which indicated the reliability of the dual RNA-seq results.

### Immune responses

**Fig. 2:**
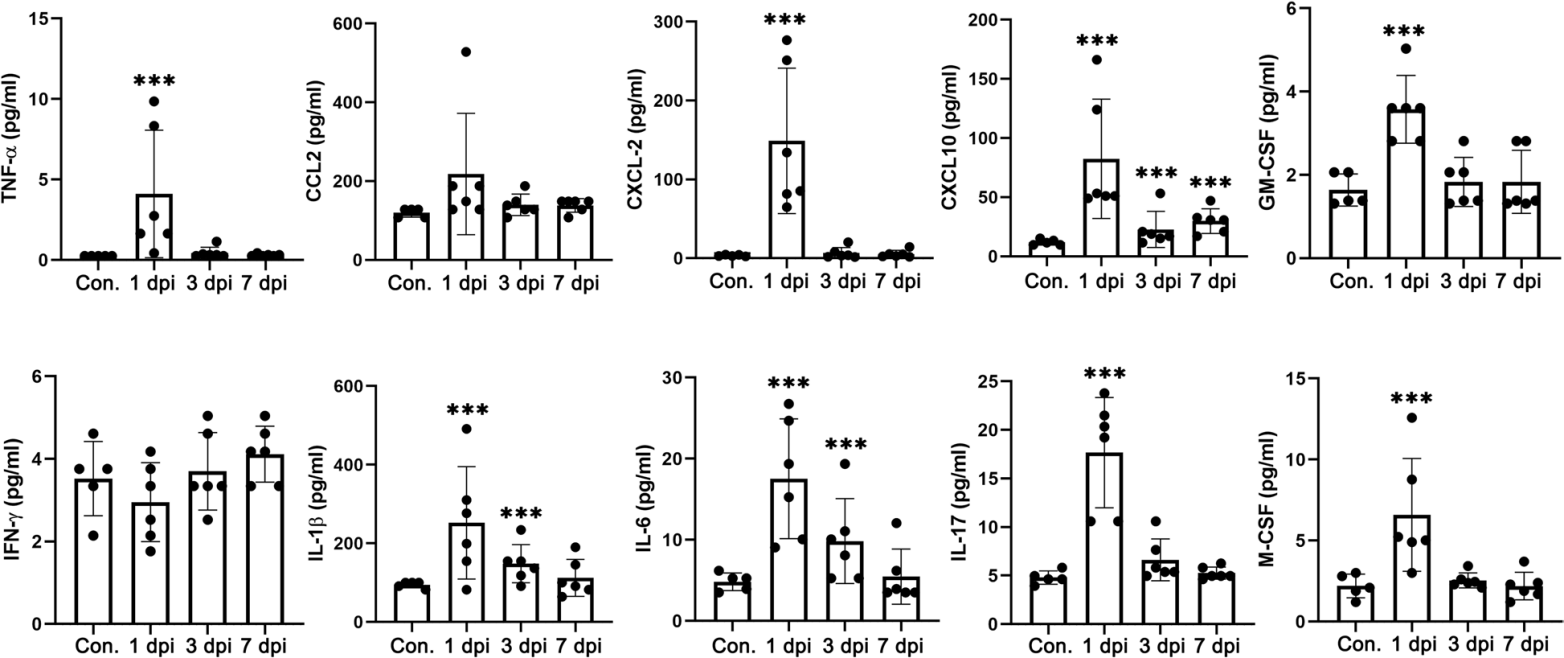
Cytokine/chemokine production in the lungs of mice induced following *N. farcinica* infection. Mice were infected with *N. farcinica* intranasally, and immune factors secreted in the lung were analyzed at different times. Con., Mice were stimulated with PBS as a control group (Student’s two-tailed *T* test, ***p<0.005, error bars represent SD)

Inflammation is a hallmark of *Nocardia* infection, which is mainly mediated by cytokines and chemokines. Notably, KEGG enrichment analysis of the DEGs during infection showed an enrichment of multiple immune-related signaling pathways of the host, including the tumor necrosis factor (TNF) signaling pathway and mitogen-activated protein kinase (MAPK) signaling pathway. To further clarify the characteristics of lung inflammation caused by *Nocardia* infection in vivo, *N. farcinica* in the exponential phase was used to infect mice intranasally, and immune factors in the lung were detected at 1, 3, and 7 days post-infection (dpi). As shown in Fig. 2, CXCL2, CXCL10, GM-CSF, M-CSF, IL-1β, IL-6, IL-17, and TNF-α were upregulated during early *N. farcinica* infection. The early host response and bacterial clearance mainly rely on neutrophils during pulmonary nocardiosis. IL-17 is involved in neutrophil infiltration during pulmonary nocardiosis [16], and the expression of IL-17 is upregulated during *Nocardia* infection. CXCL2, which has potent neutrophil chemotactic activity, was rapidly and significantly upregulated in the lung within 24 h of infection, which indicated that neutrophils recruited by CXCL2 played an important role during pulmonary nocardiosis. However, many chemokines have not yet been reported in the context of *Nocardia* infection. The production of CXCL10, which has mononuclear cell chemotactic activity, was also upregulated during early infection in this study. Interestingly, the expression of CCL2 was downregulated during lung infection caused by *N. farcinica*, which supported the differential regulation and functions of chemokines during *Nocardia* infection. GM-CSF expression was increased in the lung. Interestingly, anti-GM-CSF autoantibodies have been reported in patients with a primary cerebral abscess caused by *Nocardia* infection, and these patients may be at risk for later development of pulmonary alveolar proteinosis or other opportunistic infections [17, 18]. The expression of IFN-γ was not upregulated significantly during infection, indicating that the adaptive immunity of the Th1 response was not involved in pulmonary nocardiosis.

### *N. farcinica* Responses upon Infection of A549 cells

(i) Bacterial transcriptional adaptation at intermediate-late infection

**Fig. 3:**
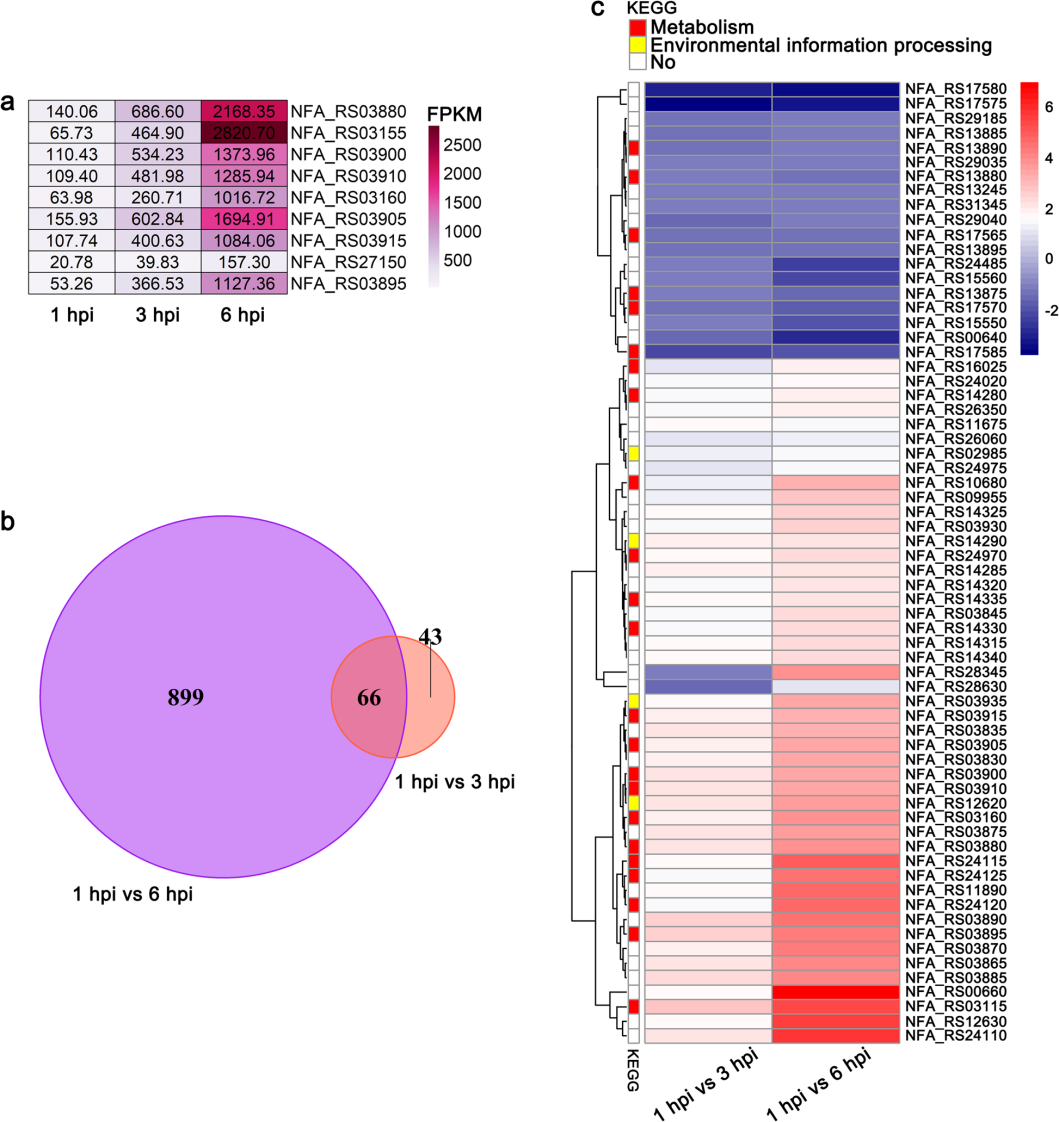
Transcriptional reprogramming of *N. farcinica* for adaptation to infection. **a** Heatmap of the enriched genes involved in the biosynthesis of siderophore group nonribosomal peptides identified by KEGG pathway analysis. No: Unknown KEGG pathway. **b** Venn diagram illustrating the number of DEGs of *N. farcinica* at 6 and 3 hpi compared with 1 hpi (log_2_-fold change ≥ 1, adjusted *P* value ≤ 0.05). The number of genes was 965 at 6 hpi compared with 1 hpi and 109 at 3 hpi compared with 1 hpi, and the number of common DEGs was 66. **c** Heatmap of selected bacterial transcripts (66 DEGs) that differentially expressed both at 3 and 6 hpi

An increased number of *Nocardia* invaded A549 cells during infection under the present experimental conditions. To gain insights into the intermediate-late transcriptome signatures after colonization, DEGs at 3 and 6 phi were analyzed and compared with those at 1 hpi (Additional file 1: Fig. S5 and Additional file 2). The biosynthesis pathway of siderophore group nonribosomal peptides was significantly enriched at both 3 and 6 hpi compared with 1 hpi. However, no studies have been conducted on the virulence of these genes in *Nocardia*. In the present experiment, the *RS03880* (*nbtG*), *RS03155* (*nbtS*), *RS03900* (*nbtC*), *RS03910* (*nbtE*), *RS03160* (*nbtT*), *RS03905* (*nbtD*), *RS03915* (*nbtF*), *RS27150* (*NFA_54680*), and *RS03895* (*nbtB*) genes, which are related to the biosynthesis of siderophore group nonribosomal peptides, were upregulated significantly (Fig. 3a). In addition, the adaptation of pathogen metabolism to the nutrients available in the host is an important prerequisite for survival [19]. These *fruA* and *fruB* genes, which are important in the metabolism of fructose, were upregulated during *Nocardia* infection. Numerous amino acids, such as proline and arginine, are available in vivo and could serve as a source of energy under anaerobic conditions. The *mftE* and *pruA* genes were upregulated during *Nocardia* infection and participated in the metabolism of arginine and proline. The pyruvate metabolism pathway was also enriched during infection, which supported the adaptation of *Nocardia* metabolism to the energy stresses present under in vivo conditions (Additional file 1: Fig. S6).

(ii) Potential key factors for bacterial survival during infection

To discover potential virulence-related genes associated with persistent infection after colonization, we first analyzed the DEGs that were expressed at both 3 and 6 hpi compared with 1 hpi (Fig. 3b). It was found 66 common genes were differentially expressed at both 6 and 3 hpi. Then, we analyzed 66 common genes and found that they were mainly involved in metabolic and environmental information processing pathways (Fig. 3c). These common DEGs are related to the biosynthesis pathway of siderophore group nonribosomal peptides, ABC transporters, and microbial metabolism in diverse environments, the phosphotransferase system (PTS), and the biosynthesis of secondary metabolites, which is crucial for the ability of *Nocardia* to cause infection and survive in the internal environment.

(iii) Confirmation of virulence genes during infection

**Fig. 4:**
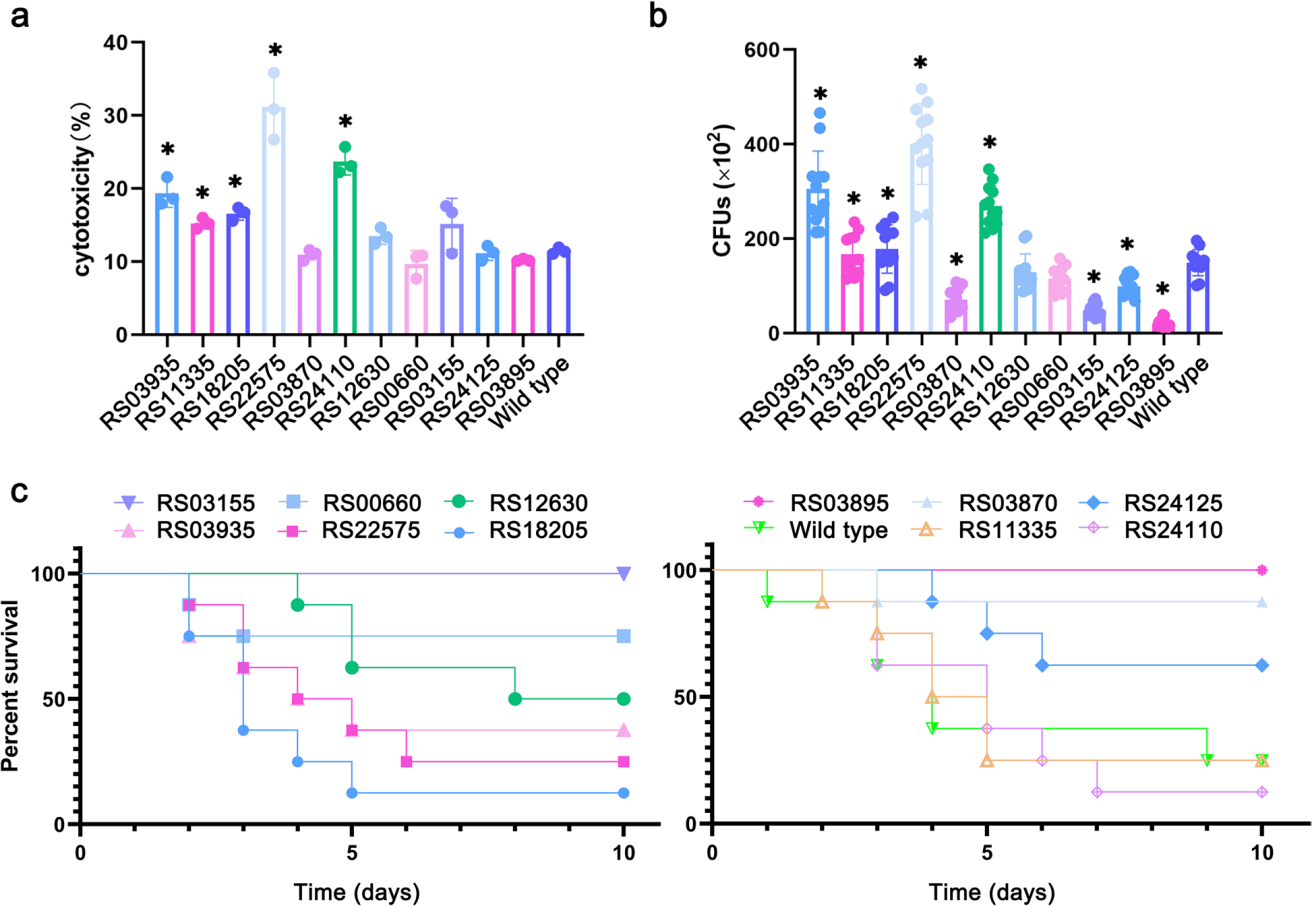
Confirmation of key virulence factors of *N. farcinica*. **a** Cytotoxicity of wild-type and mutant strains to A549 cells was detected. A549 cells were infected with bacteria at an MOI of 10:1 for 8 h, and the culture supernatant was used to detect the LDH content (Student’s two-tailed *T* test, *p<0.05, error bars represent SD). **b** The bacterial load in the lungs of mice infected with wild-type and mutant strains 2 days post-infection. Mice were infected with bacteria intravenously at a dose of 5×10^6^ CFU in 100 μl PBS, lungs were separated, and bacterial colonies in the lungs were counted 2 days after infection (Student’s two-tailed *T* test, *p<0.05, error bars represent SD). **c** Mice were intravenously infected with wild-type or mutant *Nocardia* at a dose of 3×10^7^ CFU in 100 μl PBS, and the survival of mice was monitored for 10 days

At present, there are few studies on *Nocardia* virulence factors, and many potential virulence factors have not yet been discovered. To clarify the potential virulence-related or potentially important genes that cause persistent infection, we constructed a series of genetic deletions of *N. farcinica* to confirm the functions of these genes during infection in the mouse infection model [20] (Additional file 1: Fig. S7A). We first detected lactate dehydrogenase (LDH) in the culture supernatant of A549 cells at 8 h after infection with *N. farcinica* and the mutants. As shown in Fig. 4a, the Δ*RS22575* (*narI*) and Δ*RS24110* (*NFA_48610*) mutants showed significantly higher cytotoxicity to A549 cells than the wild-type, which indicated that these genes might have potential protective effects on host cells.

To further clarify the function of these possible key genes, we infected mice and analyzed the bacterial load in the lung tissues and the mortality of the mice after infection. As shown in Fig. 4b and c, after infection with the Δ*nbtB*, Δ*nbtS*, Δ*RS00660* (*NFA_1310*), and Δ*RS03870* (*NFA_7590*) strains, the bacterial load in the lungs of mice was significantly reduced, and the survival rate was significantly improved compared with that of the mice infected with wild-type. In particular, strains Δ*nbtB* and Δ*nbtS* did not cause death of the mice, and thus, they may be the key virulence factors for *Nocardia* during infection.

Interestingly, the mice infected with *N. farcinica* showed significant behavioral changes, such as turning in circles and regressive and other neurological symptoms, but the Δ*nbtB* and Δ*nbtS* mutants did not cause behavioral changes, which revealed that *nbtB* and *nbtS* played a crucial role during *Nocardia* infection and that these genes were required for the virulence of *N. farcinica*. We also found that *NFA_7590*, which encodes siderophore-interacting protein, was upregulated, and deletion of this gene resulted in significantly impaired bacterial virulence. In addition, the ΔRS24125(*NFA_48640*) and the Δ*NFA_*1310 mutant showed attenuated virulence. Furthermore, we found that the Δ*narI*, Δ*NFA_48610*, and ΔRS03935 (*NFA_7720*) mutants failed to significantly affect the survival rate of mice compared with the wild-type after infection.

### *N. farcinica*-induced remodeling of gene expression in A549 cells

(i) Host transcriptional response upon infection at intermediate-late infection

**Fig. 5:**
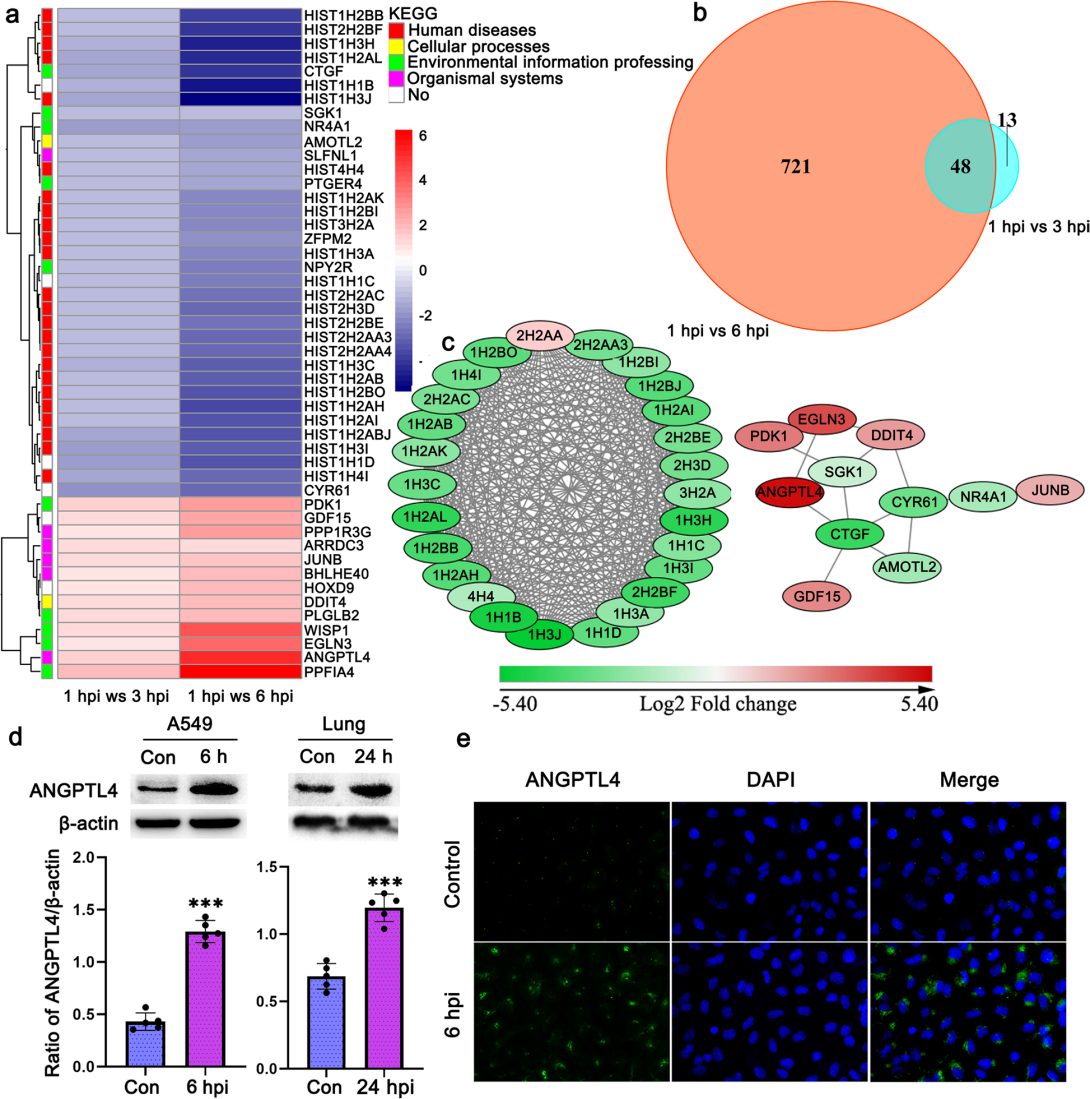
Transcriptional reprogramming of the A549 cell response to *N. farcinica*. **a** Heatmap of the 48 DEGs of A549 cells at 6 and 3 hpi compared with 1 hpi. No: Unknown KEGG pathway. **b** Venn diagram illustrating the number of DEGs of A549 cells at 6 and 3 hpi compared with 1 hpi (log_2_-fold change ≥ 1, adjusted *P* value ≤ 0.05). The number of DEGs was 769 at 6 hpi compared with 1 hpi and 61 at 3 hpi compared with 1 hpi, and the number of common DEGs in both group was 48. **c** The protein–protein interactions of 48 DEGs were analyzed using Cytoscape software. **d** The expression of ANGPTL4 was upregulated during infection in vitro and in vivo. In vitro, A549 cells were infected with *N. farcinica* at a MOI of 10 for 6 h, and the protein was analyzed by WB. In vivo, mice were infected intranasally with a dose of 5×10^6^ CFU in 50 μl PBS, and the protein in the lung was collected 24 h post-infection and analyzed using WB, each protein sample came from a different mouse (*n* = 5, Student’s two-tailed *T* test, ***p<0.001, error bars represent SD). **e** Immunofluorescence analysis of ANGPTL4 expression in A549 cells at 6 h post-infection. A549 cells were infected with *N. farcinica* at a MOI of 10 for 6 h, and then the expression of ANGPTL4 was detected

We then mainly focused on characterizing the host cell response to *Nocardia* infection (Fig. 5b, Additional file 1: Fig. S8, and Additional file 3). There were 48 DEGs during the intermediate-late stage, and further heatmaps and protein–protein interaction networks showed that these genes were mainly involved in epigenetic modifications mediated by histones (Fig. 5a, c). The expression of most histones in this experiment was downregulated during infection. Histones, primarily known for the role of condensing chromosomal DNA of mammals, are also involved in innate immune responses to antipathogens and the regulation of gene expression. Extracellular histones can activate proinflammatory signaling via Toll-like receptors [21]. *M. tuberculosis* can secrete several factors to target histones during infection, which could contribute to sustained bacterial survival in the host via histone modification [22, 23]. Indeed, modification of epigenomic processes is of importance for bacterial pathogen infection, and regulating or inhibiting these processes via histones may alter the outcome of infection.

Angiopoietin-like 4 (ANGPTL4) was upregulated significantly at 6 hpi compared with 1 hpi. ANGPTL4 expression was reported to be elevated and involved in lung damage during infection caused by numerous stimuli, such as influenza pneumonia [24, 25]. Pneumonia is a common clinical symptom caused by *Nocardia* infection, and elevated ANGPTL4 may be involved in the lung damage caused by *Nocardia*. As shown in Fig. 5d and e, the production of ANGPTL4 was elevated significantly at 6 h after infection in A549 cells. In addition, the ANGPTL4 protein in the lungs of mice intranasally infected by *N. farcinica* was significantly upregulated. However, the mechanism by which ANGPTL4 mediates pulmonary inflammation induced by *Nocardia* is unclear and requires further study.

In addition, ANGPTL4 plays an important role in regulating the integrity of endothelial vascular junctions via integrin pathways and destroys claudin-5 clusters and intercellular VE-cadherin [26]. Liu et al. reported that ANGPTL4 was significantly upregulated in meningitis and induced an increased permeability of the blood–brain barrier (BBB) by elevating myosin light chain 5 (MYL5) expression through RhoA signaling pathway activation [27]. The first step of infection of the brain induced by *Nocardia* is BBB disruption, but the underlying mechanism is unclear. In this study, ANGPTL4, which is involved in BBB integrity, was significantly upregulated, which indicated that this gene might play a critical role in the BBB destruction induced by *Nocardia* infection.

WNT-inducible signaling pathway protein-1 (WISP1), which plays an important role in lung injury, was significantly upregulated during *Nocardia* infection. Recently, WISP1 was shown to promote the inflammatory response via TLR4/CD14 pathways in sepsis-induced lung injury [28]. DNA damage-inducible transcript 4 (DDIT4), encoding Rtp801, is also involved in inflammation in the lung and can promote alveolar inflammation and apoptosis of alveolar cells by suppressing mTOR signaling pathways, leading to lung injury [29]. In our study, we found that the expression of DDIT4 was upregulated, which suggested that this gene might participate in lung inflammation induced by *Nocardia* infection.

(ii) Neurodegenerative symptom analysis

**Fig. 6:**
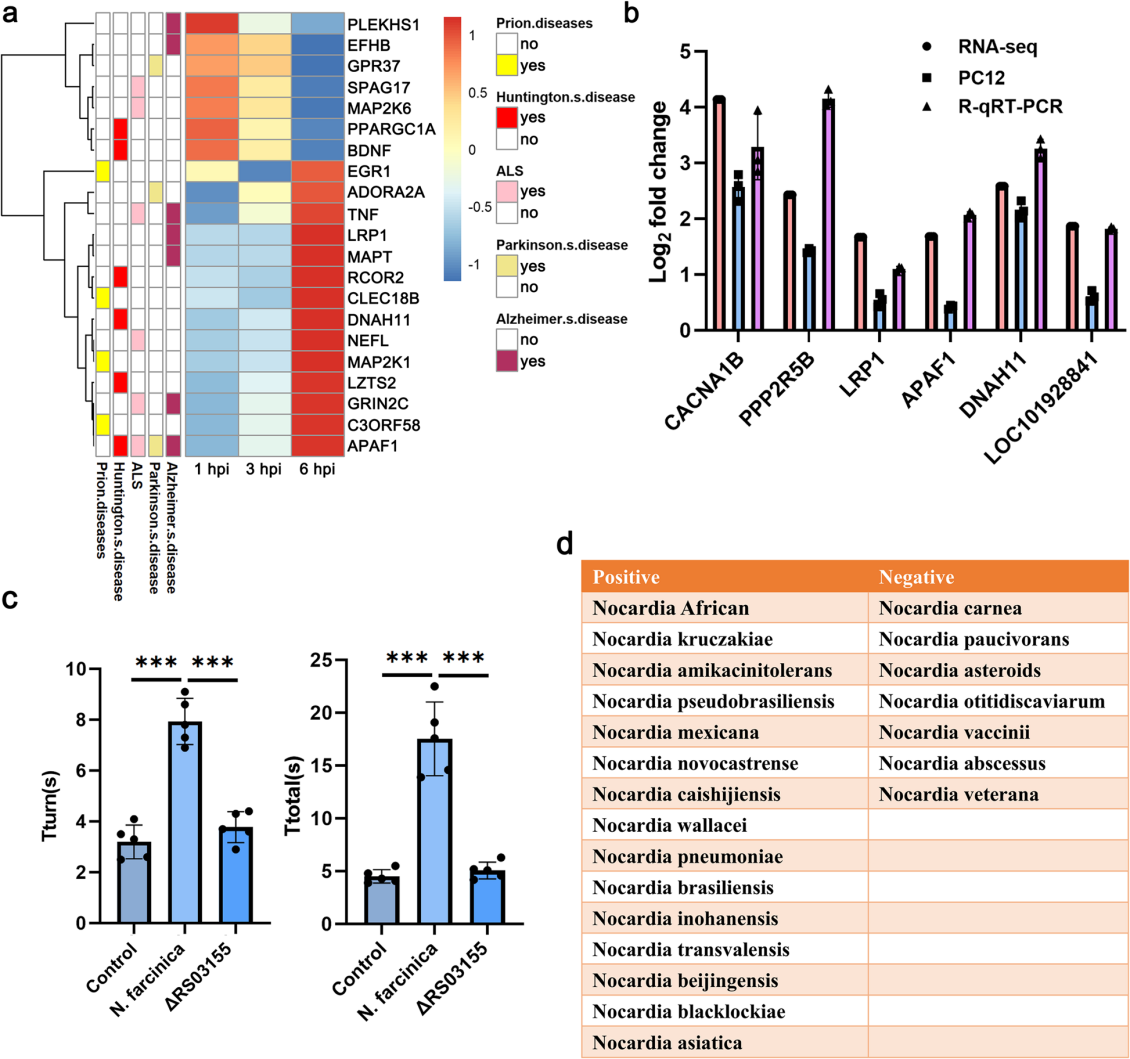
*Nocardia* infection involved in neurological behavioral disorder. **a** Heatmap of the DEGs related to neurodegenerative diseases. Yes: Genes were included; No: Genes were not included. **b** The genes involved in neurodegenerative diseases were verified by qRT–PCR. RNA-seq, the data for dual RNA-seq. R-qRT–PCR, RNA samples from repeated experiments. PC12, RNA samples were extracted from PC12 cells infected with *N. farcinica* at a MOI of 10 for 6 h. **c** The time for the mice to turn completely downward (Tturn) and reach to the floor was recorded (*n* = 5 mice per group, *N* = 3 replicates per mouse, Student’s two-tailed *T* test, ***p<0.001, error bars represent SEM). **d** Clinical strains of *Nocardia* related to neurological behavioral disorder. Twenty-two clinical strains of *Nocardia* were analyzed, and 15 strain infections showed neurological behavioral disorders, and 7 strain infections showed few obvious neurological behavioral symptoms

During the in-depth exploration and analysis of the KEGG pathway, we found that some DEGs related to the pathways of the nervous system and neurodegenerative diseases were dysregulated, which caught our attention. *Nocardia* can quickly cross the blood–brain barrier and enter the brain parenchyma, causing central nervous system infection. Under our experimental conditions, not only were PD-related genes differentially expressed, but genes related to other nervous system and neurodegenerative diseases, such as Alzheimer’s disease (AD), amyotrophic lateral sclerosis (ALS), and other related genes, were also differentially expressed, which indicated that *Nocardia* entered the brain parenchyma and might cause a variety of symptoms related to the nervous system or neurodegenerative diseases (Fig. 6a). As shown in Fig. 6b, the RT–PCR results from PC12 cells were consistent with the gene expression trend of the RNA sequencing results.

To further verify the association between *Nocardia* infection and PD-like symptoms through in vivo experiments, we infected mice with *N. farcinica* intravenously and analyzed the symptoms after infection. We found that all mice showed neurological symptoms without death post-infection with 5 × 10^6^ CFU *N. farcinica*. However, a higher infectious dose caused some mice to die, and a lower infectious dose resulted in insignificant neurological symptoms in some mice. Therefore, an infection dose of 5 × 10^6^ CFU was used in the subsequent study. The pole test, a method for testing the motor performance in PD, was applied to evaluating the motor dysfunction caused by *Nocardia* infection. As shown in Fig. 6c, *N. farcinica* infection caused motor dysfunction as measured by prolonging in the Tturn and Ttotal time as compared to control group. We also found that the Δ*nbtS* mutant strain almost have no effect on Tturn and Ttotal time as compared to control group. Besides, the behavioral disorder was visible as follows: (a) head falling on one side (Additional file 4: video 1); (b) a tendency to turn in the same direction when lifted by the tail (Additional file 4: video 2); (c) body quiescent tremor and rhythmical and vertical head movements (Additional file 4: video 1); (d) stagnation and turning backward in the same direction in unfamiliar environments, with the hind limbs open and stride length altered (Additional file 4: video 3); (e) circling of some mice at 3 months after infection (Additional file 4: video 4). The above symptoms further confirmed that *N. farcinica* infection could cause a series of neurodegenerative-like disease symptoms, which indicated that *Nocardia* infection might be involved in the development of neurodegenerative diseases such as PD.

However, can all *Nocardia* infections invade the brain and cause neurological symptoms? We analyzed the relationship between 22 clinical *Nocardia* infections (Additional file 1: Fig. S9) and neurodegenerative diseases. Mice were infected intravenously with the above *Nocardia* strains, and symptoms were observed after infection. We found that *N. Africa*, *N. kruczakiae*, *N. amikacinitolerans*, *N. pseudobrasiliensis*, *N. mexicana*, *N. novocastrense*, *N. caishijiensis*, *N. wallacei*, *N. pneumoniae*, *N. brasiliensis*, *N. inohanensis*, *N. transvalensis*, *N. beijingensis*, *N. blacklockiae*, and *N. asiatica* infection caused neurological behavioral disorder at different times after infection, and the other strains did not cause obvious behavioral disorder symptoms after infection under the conditions used in our experiment (Fig. 6d). These results indicated that not all *Nocardia* infections could cause neurological symptoms, and strains capable of inducing neurological infection should arouse attention in clinical work.

(iii) Microglial activation mediates the development of PD-like symptoms induced by *Nocardia*

**Fig. 7:**
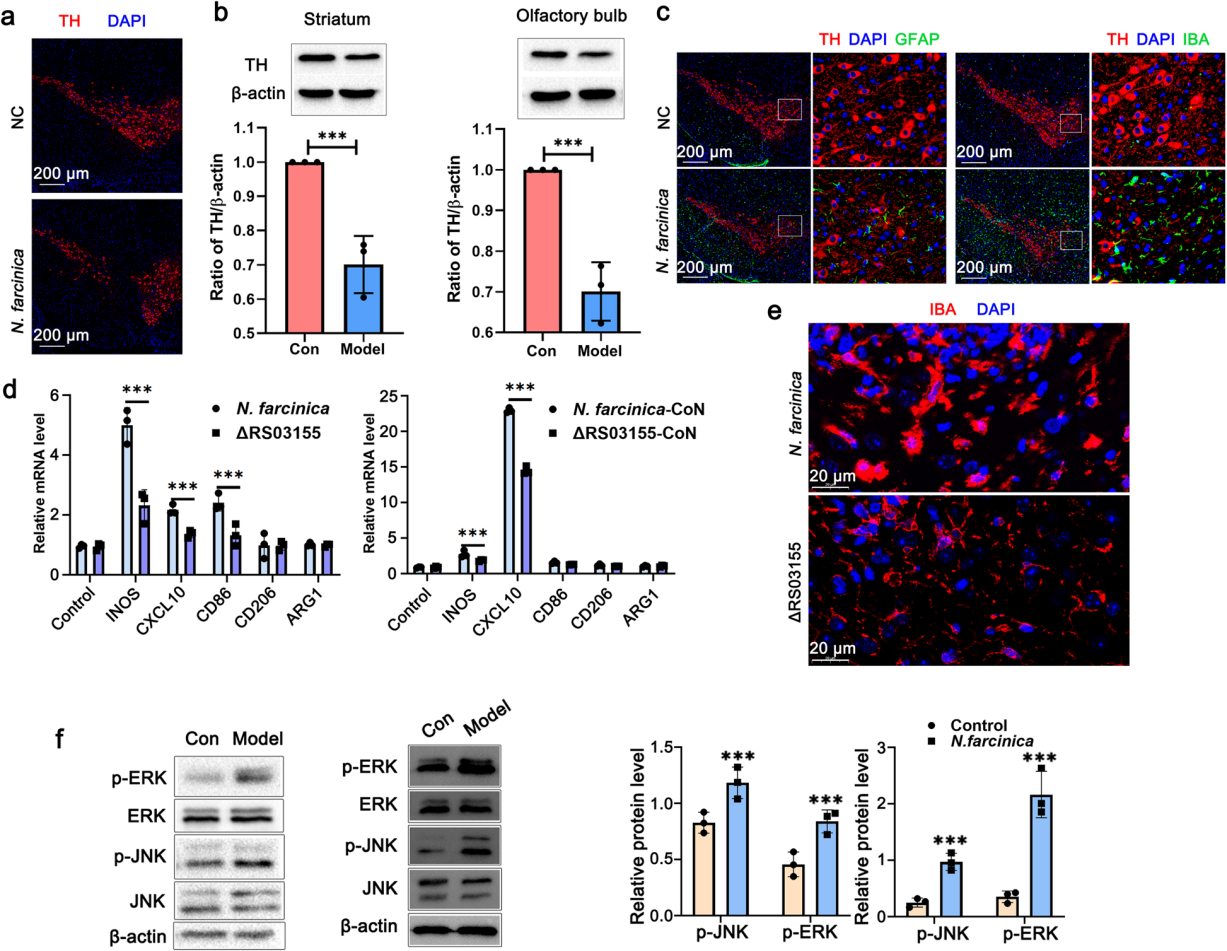
Characteristics of neuroinflammation induced by *N. farcinica*. **a** The number of dopaminergic neurons in the substantia sections of mice was detected after infection with *N. farcinica*. Mice were infected intravenously at a dose of 5 × 10^6^ CFU, and the substantia nigra sections from mice were analyzed by immunofluorescence for TH expression levels. **b** TH contents in the striatum and olfactory bulb of the mouse brain were analyzed after infection with *N. farcinica*. The striatum and olfactory bulb extracts of mice were analyzed for TH using WB, each protein sample came from a different mouse (*n*=3 biological replicates per group, Student’s two-tailed *T* test, ***p<0.01, error bars represent SD). **c** The substantia nigra sections of mice post-infection with *N. farcinica* were analyzed by immunofluorescence for microglia and astrocytes. **d** The mRNA expression of phenotypic markers in BV2 cells was analyzed after stimulation with *N. farcinica*, ΔRS03155, or CoN. BV2 cells were infected with *N. farcinica* or ΔRS03155 for 2 h, total RNA was extracted, and the expression of M1-like markers (INOS, CXCL10, and CD86) and M2-like markers (CD206 and ARG1) was analyzed using RT–PCR. BV2 cells were stimulated with CoN for 4 h, and phenotypic markers were analyzed using RT–PCR (Student’s two-tailed *T* test, ***p<0.01, error bars represent SEM). **e** Brain sections from mice were analyzed by immunofluorescence to assess the microglial morphology after infection with *N. farcinica* or ΔRS03155. **f** Activation of JNK and ERK was analyzed in BV2 cells (left) and the striatum (right) of mice post-infection with *N. farcinica*. BV2 cells were infected with *N. farcinica* at a MOI of 10 for 6 h, and the proteins were extracted and analyzed. Protein was extracted from the striatum of mice infected with *N. farcinica* for 7 days and analyzed for JNK and ERK by WB, each protein sample came from a different mouse (*n* = 3 biological replicates per group, Student’s two-tailed *T* test, ***p<0.001, error bars represent SD)

Damage or loss of dopaminergic neurons in the substantia nigra and a decreased dopamine content in the striatum are typical pathological features of PD. As shown in Fig. 7a, after *Nocardia* infection, the number of dopaminergic neurons in the substantia nigra of the mouse brain was significantly reduced post-infection, and the shape of the dopaminergic neurons was irregular. To further validate whether *Nocardia* infection caused PD-like symptoms, we analyzed the changes in dopamine content in the striatum of the mouse brain. As shown in Fig. 7b, the tyrosine hydroxylase (TH) in the striatum of the mouse brain was significantly reduced after infection, further indicating that *Nocardia* infection could cause a decrease in striatal dopamine content, which in turn led to PD-like neurological symptoms. In addition, we also observed a decrease in dopamine content in the olfactory bulb (Fig. 7)b.

It has been reported that neuroinflammation is associated with neurodegenerative diseases and that microglia play a key role in inflammatory responses in the central nervous system [30–32]. In the present study, we found that microglia in the substantia nigra region were significantly activated, while astrocytes were not activated (Fig. 7c). Activated microglia can be divided into two different types: M1-like microglia and M2-like microglia [33]. However, whether *Nocardia* infection can cause polarization of microglia remains unclear. Therefore, we detected the expression of markers of M1 (iNOS, CXCL-10, and CD86) and M2 (CD206 and ARG1) microglia after *Nocardia* infection using BV2 cells (Additional file 1: Fig. S7B). As shown in Fig. 7d, M1-type markers (iNOS, CXCL-10, and CD86) were significantly upregulated after infection, and the expression level of M2-type markers (CD206 and ARG1) did not change significantly. In addition, we also found that conditioned medium from *N. farcinica*-infected (CoN) RAW264.7 cells could significantly stimulate the expression of M1 markers (iNOS and CXCL-10) in microglia. This result indicated that *Nocardia* infection might cause PD-like symptoms via neuroinflammation mediated by polarized M1 microglia. In addition, both *Nocardia*-microglia and macrophage-microglia interactions played a crucial role in driving M1 microglia polarization.

To further clarify the characteristics of neuroinflammation caused by *Nocardia* infection, we analyzed inflammatory factors in the brain tissue of mice after *Nocardia* infection. To analyze whether the inflammatory factors in the brain were secreted by cells in the brain or were derived from peripheral blood and to analyze the relationship between the inflammatory factors in peripheral blood and brain tissue, we also detected the cytokine content in peripheral blood during different infection periods. As shown in Additional file 1: Fig. S10, cytokines in the brain (TNF-α, CCL-2, CXCL-2, CXCL-10, IL-1β, IL-6, IL-17, and M-CSF) were upregulated after infection at 3 dpi, and the cytokine content gradually decreased with extension of the infection time. In particular, the upregulated expression of CCL-2, CXCL-2, and CXCL-10 was the most significant. It has been reported that CXCL10, secreted from M1-type activated microglia, plays a crucial role in stimulating Th1 cell infiltration by serving as the ligand for CXCR3 T cells [34]. Interestingly, we found that CoN could stimulate microglia to significantly upregulate CXCL-10 compared with the direct interaction of *N. farcinica* and microglia. This result suggested that the upregulated CXCL-10 in the brain was partly secreted by polarized microglia, which was mainly mediated through macrophage-microglia interactions (Fig. 7d). Although these cytokines were upregulated in peripheral blood, their concentrations were significantly lower than those in brain tissue, which indicated that these cytokines were secreted by immune cells in the brain. In addition, the expression of IL-1β was significantly elevated at 3 dpi and lasted until 7 dpi in the brain. It has been reported that microglia activated by lipopolysaccharide (LPS) cause dopaminergic neuron damage in an IL-1β-dependent manner, resulting in PD-like neurological behavioral disorder [35].

To further analyze the mechanism of *Nocardia* infection responsible for PD-like neurological symptoms, the Δ*nbtS* mutant strain, which does not cause neurological symptoms after infection, was used in the next study. The mutant strain stimulated BV2 cells to produce fewer inflammatory factors, such as iNOS and CXCL-10, than the wild-type strain after infection. In addition, conditioned medium from the wild-type strain promoted BV2 cells to express more inflammatory factors than medium from the Δ*nbtS* mutant strain, especially CXCL-10 (Fig. 7d). These results indicated that CXCL-10 or iNOS played an important role in the neuroinflammation-mediated nervous system symptoms caused by *Nocardia* infection.

In vivo, we found that the morphology of microglia was significantly different in the brain after infection with the wild-type and Δ*nbtS* mutant strains. As shown in Fig. 7e, the main morphology of brain microglia after infection by the mutant strain had a more rod-like and amoeboid shape, while the microglia mainly had a ramified phenotype after infection with the Δ*nbtS* mutant strain. These results indicated that the activation of microglial status played a key role in the neurological symptoms induced by *Nocardia* infection.

Continuous activation of microglia is linked to the progression of PD by inducing dopaminergic neuron degeneration [36]. The MAPK signaling pathway in microglia plays an important role in the progression of PD [37]. In the present study, we found that *Nocardia* infection could cause significant activation of microglia, so we further studied the activation status of inflammation-related signaling pathways in vivo and in vitro. As shown in Fig. 7f, the extracellular regulated protein kinases (ERK) and c-jun n-terminal kinase (JNK) pathways were activated in BV2 cells after infection with *Nocardia*. In addition, these signaling molecules were also phosphorylated in the striatum of mouse brains infected with *Nocardia*. These results indicated that *Nocardia-*induced neurological behavioral disorder by activating microglia through the MAPK signaling pathway.

## Discussion

With the improvement of diagnostic technology, an increasing number of cases of nocardiosis have been reported. However, research on *Nocardia* infection is still insufficient. Several studies have applied a dual RNA-seq approach to reveal simultaneous transcription adaptation in host cells and pathogens [14, 15, 38, 39]. Here, we applied dual RNA-seq to obtain transcriptome data at different time points post-infection, confirmed several novel virulence genes for *Nocardia* infection in vivo, and expounded on the possible mechanism by which *Nocardia* infection causes a series of neurological symptoms, such as PD-like symptoms.

In our study, we found that the differential expression of genes related to iron uptake was the most significant. Using gene knockout approach, we verified *nbtB* and *nbtS* were essential for *Nocardia* infection. This is the first study to report and verify *nbtB* and *nbtS* as key virulence factors for *Nocardia* in vivo. Indeed, the genes involved in iron acquisition and transport in *Nocardia* are still unclear, and further analysis of these genes related to iron uptake is essential for elucidating the pathogenic mechanism of *Nocardia*. Histones play a critical role in regulating gene expression by binding to DNA. In addition, histones involved in inflammatory responses are an important component in the recruitment of neutrophils to kill bacteria [40]. It has been reported that H2A and H2B have the capacity to neutralize endotoxins and act as antimicrobials against *Escherichia coli* [41]. Bacteria have also evolved to resist killing by histones. *Finegoldia magna* can bind histones with surface proteins and degrade histones with secreted proteases [42]. In this study, we found that histones were downregulated significantly at both 6 and 3 hpi (Fig. 5a, c). However, the role of dysregulated histones in the host cell response to *Nocardia* infection has not been reported, and it is necessary to further clarify the functions of histones in nocardiosis.

Though several studies have documented that *Nocardia* infection can lead to PD-like neurological symptoms, there is some controversy about the relationship of *Nocardia* infection to the occurrence and development of PD [13, 43, 44]. We confirmed that *N. farcinica* infection was able to induce PD-like symptoms (Additional file 4: video 1, 2, and 3). It has been reported that *Nocardia* can change to cell wall-deficient L-forms after invading macrophages, which may be the reason why *Nocardia* is difficult to culture [45]. The L-forms of *Nocardia* will further increase the difficulty of diagnosing nocardiosis. Therefore, it is a challenge to diagnose patients with clinical PD-like symptoms caused by *Nocardia* infection, thus weakening the potential association between *Nocardia* and PD or PD-like disease, which should attract the attention of clinicians. Corrales et al. found an inflammatory response in parts of the brain in which *Nocardia* was not detected post-infection [46]. Similar to this finding, we noted the occurrence of diffuse inflammation in the brain, even in areas that were not affected by *Nocardia*, and this inflammation persisted even if the bacteria could not be cultured. However, the mechanism is currently unclear and needs to be further resolved in the future. Next, we found that only a portion of the *Nocardia* were capable of causing neurological symptoms, which indicated the presence of differences in brain susceptibility between *Nocardia* species and that the mechanism mediating the differences in susceptibility requires further study. These findings further remind us to focus on clinical strains of *Nocardia* to which the brain is susceptible.

Microglial activation-mediated neuroinflammation plays a key role in central nervous system diseases, such as PD. Here, we found that microglia but not astrocytes were activated after infection with *N. farcinica* (Fig. 7c), indicating the key role of microglial activation in neuroinflammation caused by *N. farcinica*. Further study showed that *N. farcinica* could drive M1 microglial polarization directly or indirectly through macrophages. Interestingly, we found that the neurological symptoms were completely abolished after infection with the Δ*nbtS* mutant strain, and the expression of inflammatory factors, such as iNOS and CXCL-10, in BV2 cells stimulated with this mutant strain was lower than that induced by the wild-type strain after infection. Finally, we found that *N. farcinica* could stimulate the MAPK pathway of innate immunity both in vitro and in vivo, which suggested that the MAPK pathway might be involved in the activation of microglia and lead to neurological behavioral disorder after *Nocardia* infection.

Indeed, our study does have certain limitations. Is the neurological symptom caused by direct infection of neurons by the *Nocardia* or is it mainly mediated by neuroinflammation mediated by microglia? This issue needs to be further investigated. In addition, whether the virulence gene *nbtS* are secreted to exert virulence has not yet been studied, and the pathogenic mechanism is unclear. Finally, transcriptome analysis of *Nocardia*-infected brain tissue will helps to clarify the molecular mechanism of *Nocardia*-induced neurological symptoms.

## Conclusions

In summary, we disclosed a series of novel virulence genes and metabolic pathways for *Nocardia* and clarified the relationship between *Nocardia* infection and neurological diseases, especially PD-like symptoms. Our findings provide insight into a deeper understanding of host-pathogen interactions for emerging or neglected *Nocardia* and will lay the foundation for future studies on the pathogenesis of *Nocardia*. In addition, our study may serve as a blueprint that can be applied to other bacterial pathogens in the future.

## Methods

### Antibodies and reagents

Anti-p-JNK (Cat*#* 4668), anti-JNK (Cat*#* 9252), anti-p-ERK 1/2 (Cat*#* 4370), anti-ERK (Cat*#* 4695), and anti-β-actin (Cat*#* 3700) were purchased from Cell Signaling Technology. Anti-tyrosine hydroxylase (Cat*#* 75875) was purchased from Abcam. Anti-ANGPTL4 (Cat*#* 67577) was purchased from Proteintech. Anti-Iba1 (Cat*#* GB13105-1) and anti-GFAP (Cat*#* GB11096) antibodies were purchased from Servicebio. Luminex Mouse Magnetic Assay (Cat*#* LXSAMSM-11) was purchased from R&D Systems. Cytotoxicity Assay (Cat*#* G1782) was from Promega.

### Cell line and Nocardia culture

Cells were grown in DMEM supplemented with 10% fetal calf serum (FCS; TransGen Biotech), 2 mM l-glutamine (Gibco), and 1 mM sodium pyruvate (Gibco) in T-75 flasks (Corning) in a 5% CO_2_ humidified atmosphere at 37 °C. *Nocardia* strains were grown in brain heart infusion medium (BHI, Oxoid) at 37 °C.

### Infection assay

In vitro, 1.5×10^6^ cells/well were seeded in six-well plates. The fresh bacteria were resuspended in complete DMEM to prepare the inoculum. Infections were performed at a multiplicity of infection (MOI) of 10. The inocula or medium-only controls were added to the apical surface of the cultures and incubated for 1 h in a 5% (v/v) CO_2_ humidified atmosphere at 37 °C. The cells were washed three times with PBS to remove the free-floating *N. farcinica* prior to RNA extraction.

### Isolation of total RNA from infected cells

Total RNA was isolated at 1, 3, and 6 h from *N. farcinica*-infected A549 cells and corresponding mock controls (three replicates per time point). Total RNA isolated from bacteria conditioned in infection medium at 37 °C in 5% CO_2_ for 1 h was used as a bacterial baseline control. Three technical replicates (individual wells) were pooled into one biological replicate. Three biological replicates were used. Before isolation, the wells were gently rinsed three times with PBS after 1 h of infection to remove nonadherent *N. farcinica* cells, and the total RNA was extracted according to the manufacturer’s recommended protocol. The total RNA sample concentrations, RIN, and size were detected using an Agilent 2100 Bioanalyzer (Agilent RNA 6000 Nano Kit), and the purity of the samples was tested using a NanoDrop^TM^.

### cDNA library construction and dual RNA-seq

Host and bacterial ribosomal RNAs were simultaneously depleted by a 1:1 mixture of human/mouse/rat and gram-positive bacterial capture probes (Ribo-Zero rRNA Removal Kits, Illumina, USA). cDNA sequencing of the 15 samples was carried out on the Illumina HiSeq Xten platform in 75-bp paired-end mode at The Beijing Genomics Institute. For cDNA library preparation, 450 ng RNA of each sample was used. Ribodepleted RNA samples were fragmented using fragmentation reagent, and first-strand cDNA was generated using random primer reverse transcription, followed by second-strand cDNA synthesis.

### Read mapping and data analysis

Sequencing reads were filtered with SOAPnuke software (https://github.com/BGI-flexlab/SOAPnuke, version v1.5.2) to remove reads with adaptors, reads in which unknown bases (N) made up more than 10% and low-quality reads. For the transcriptome analysis, reads were aligned in paired-end mode to a human genome (hg19) and *N. farcinica* IFM10152 genome (NCBI RefSeq accession numbers: NC_006361.1, NC_006362.1, NC_006363.1) using HISAT (Hierarchical Indexing for Spliced Alignment of Transcripts, Version v2.0.4) [47] with default settings. To avoid cross mapping, before mapping to the *N. farcinica* IFM10152 genome, clean reads from infected samples and the mimic control group were aligned to the human genome, and unmapped pairs of reads were then used for alignment to the bacterial genome [15]. For mapping of the human genome, clean reads were aligned to the human genome, and those reads that were cross mapped with bacteria were discarded. Clean reads were mapped to references using *Bowtie2*(V2.2.5) [48], and then the FPKM (fragments per kilobase million) was calculated with *RSEM* (V1.2.12) [49]. Differential expression was evaluated by comparing the data from infected samples to that from mimic control samples. DEGs that were differentially expressed≧2-fold with an adjusted≦0.05 were detected with DEseq2 (V1.36.0) with default parameter as described previously [50]. Differentially expressed genes between the infected and control groups were identified using the following thresholds: |log2fold change| of ≥1 and adjusted *p* value of ≤0.05. The Kyoto Encyclopedia of Genes and Genomes (KEGG) and clustering analysis of DEGs were completed on Dr. Tom system (https://biosys.bgi.com).

### qRT–PCR

To validate the RNA-seq data, total RNA from sequenced or repeated infection samples was used in qRT–PCR. Briefly, qPCR was performed with SYBR Premix Ex Taq II reagents (TaKaRa, Japan) on a 7500 Fast Real-Time PCR System (Applied Biosystems) using 2 μl of the diluted cDNA samples, 10 μl of Power SYBR master mix, and 1 μl of 10 M gene-specific primer. The housekeeping genes *secA* and *β-actin* for *Nocardia* and epithelial cells, respectively, were used to normalize the level of gene expression. Fold changes in gene expression were determined using the 2(−∆∆Ct) method [51].

### Animal model

To obtain the minimum infectious dose for neurological symptoms, female C57BL/6J mice were infected intravenously with *N. farcinica* at approximately 1× 10^8^, 1× 10^7^, 5× 10^6^, 1× 10^6^, and 5× 10^5^ CFU in 100 μl PBS, and the number of mice with neurological symptoms was analyzed.

### Pole test

Mice were trained to climb a pole 3 days before the pole test. In test, mice were placed on the top of a rough-surfaced and vertical pole (1 mm in diameter, 60 cm in height) with head up and the time needed to turn downward (Tturn) and the time to reach the bottom (Ttotal) were recorded. Three times measurements was used as the result.

### Construction of deletion mutants

The gene in-frame deletion mutant was constructed via homologous recombination according to previously described methods [20]. Upstream DNA fragments and downstream gene fragments were amplified by PCR using primers (Additional file 1: Table S2). These two fragments were subsequently ligated to generate a gene deletion fragment, which was then cloned into the pK18mobsacB vector. *N. farcinica* in logarithmic phase was washed 3 times with ice-cold water and then resuspended in 10% ice-cold glycerol to generate competent bacteria. The recombinant pK18mobsacB plasmid was transformed into competent bacteria and incubated in BHI broth for 2 h at 37 °C. The positive colonies were first selected on BHI plates containing neomycin and then selected on BHI plates containing 20% sucrose. The deletion of genes were confirmed by PCR (Additional file 1: Table S2).

### Virulence determination

Mice were infected intravenously with wild-type or mutant *Nocardia* in logarithmic phase at a dose of 3×10^7^ CFU in 100 μl PBS, and then the survival rate of mice was analyzed after infection. Mice were infected intravenously with wild-type or mutant *Nocardia* at a dose of 5×10^6^ CFU in 100 μl PBS, and the lung tissue was separated and ground in 1 ml PBS. Then, the colonies in the lung were counted.

### Cytotoxicity assay

A549 cells were cultured in DMEM supplemented with 10% FBS at 37 °C for 16–18 h before infection. Wild-type or mutant *Nocardia* in logarithmic phase were used at a MOI of 10:1. The CytoTox 96® Non-Radioactive Cytotoxicity Assay (Promega, USA) was used to measure the cytotoxicity according to the manufacturer’s instructions at 8 h post-infection as previously described [52].

### Preparation CoN and CoU

RAW264.7 cells were infected with *N. farcinica* or Δ*nbtS* strains at a MOI of 10. Cell culture supernatants were obtained and filtered through a 0.2-μm sterile filter 24 h after infection [53]. The conditioned medium from *N. farcinica-* or mutant strain-infected cells was termed CoN, and medium from uninfected cells was used as a control. *N. farcinica* or mutant strains at a MOI of 10 or conditioned medium at a dilution of 1:3 were used to prime microglial cells for 4 h, and then total RNA was isolated and analyzed.

### Western blot analysis

BV-2 or A549 cells were seeded onto 6-well plates for 16–18 h and infected with *N. farcinica* (MOI=10). After infection, the cells were lysed with lysis buffer supplemented with phosphatase and protease inhibitors (CWBIO, China) on ice as previously described [54]. The lung, striatum, and olfactory bulb isolated from mice were added to lysis buffer and ground on ice to extract the total protein. In brief, the total protein were separated by SDS–PAGE and then transferred to polyvinylidene fluoride membranes (Millipore). The membranes were incubated with primary antibodies and then incubated with HRP-conjugated anti-rabbit IgG (Beyotime Biotechnology), and the bands were measured using a Western Lightning Plus ECL kit (PerkinElmer, USA).

### Cytokine detection

Mice were infected intranasally with *N. farcinica* (1×10^7^ CFU), and lung tissue was sampled at 1, 3, and 7 days post-infection and then ground on ice with protease inhibitors. Then, the tissue supernatant was obtained after centrifugation (10,000*g*, 10 min) and stored at −80 °C for detection. For serum and brain, mice were infected intravenously with *N. farcinica* (5×10^6^ CFU). Then, brain tissue and blood were isolated at 3, 7, and 14 days after infection. The supernatant of brain tissue and serum was collected after centrifugation and stored at −80 °C until testing. Cytokines (CCL2, CXCCL2, CXCL10, GM-CSF, IFN-γ, IL-1β, IL-2, IL-6, IL-17, M-CSF, TNF-α) in tissue supernatant and serum were determined by a Multiplex® system (R&D, USA) according to the manufacturer’s recommendations.

### Immunofluorescence staining

Mice were sacrificed 14 days post-infection and perfused intracardially with PBS and 4% paraformaldehyde. Then, the brain was collected and preserved in 4% paraformaldehyde. The coronal sections of brain (4 μm) containing the SNpc region were dissected. The sections were then incubated with primary antibodies targeting tyrosine hydroxylase, GFAP and Iba-1. After washing, the sections were treated with Cy3- or Alexa Fluor 488-conjugated secondary antibodies. The nuclei were labeled via DAPI counterstaining. Images were captured with a laser scanning confocal microscope (LSM 700, Carl Zeiss).

For cells, A549 cells were seeded into 24-well plates with glass coverslips and grown until confluence. Then, A549 cells were infected with *N. farcinica* at logarithmic phase at a MOI of 10 for 6 h. Anti-ANGPTL4 antibody was used as a primary antibody and incubated for 30 min at 37 °C. Subsequently, anti-rabbit IgG was added to the cells for 30 min at 37 °C after 3 washes with PBS. Finally, DAPI was added to the cells for 10 min and then imaged.

### Ethics statement

All animal research was performed in accordance with animal ethics guidelines and approved protocols. The animal experiments were approved by the Ethics Review Committee of Shandong Provincial Hospital.

### Statistical analysis

All data were analyzed and presented using GraphPad Prism 8. Analyses were performed using Student’s two-tailed *T* test and reported as mean ± standard deviations (SDs). Each statistical test used for each figure is described in the legends.

## Supplementary Information

**Additional file 1: Figure S1.** [Heat map of the Pearson correlation of gene expression between samples for RNA-seq data]. **Figure S2.** [KEGG pathway analysis of DEGs for N. farcinica and A549 cells]. **Figure S3.** [KEGG pathway analysis]. **Figure S4.** [Validation of RNA-seq via qRT–PCR]. **Figure S5.** [Volcano plot obtained from DESeq2 analysis of DEGs from Nocardia farcinica]. **Figure S6.** [KEGG pathway analysis of *Nocardia* at 3 and 6 hpi]. **Figure S7.** [Analysis of the *N. farcinica* mutant strains]. **Figure S8.** [Volcano plot obtained from DESeq2 analysis of DEGs from A549 cells]. **Figure S9.** [The colony status of the *Nocardia* strains on the blood plate]. **Figure S10.** [Analysis of inflammatory factors in serum and brain]. **Figure S11.** [Original gel images with indicated figures].

**Additional file 2. **DEGs of *N. farcinica* at 3 and 6 phi.

**Additional file 3.** DEGs of A549 cells at 3 and 6 phi.

**Additional file 4: Video 1.** [head falling on one side, body quiescent tremor and rhythmical and vertical head movements]. **Video 2.** [a tendency to turn in the same direction when lifted by the tail]. **Video 3.** [stagnation and turning backward in the same direction in unfamiliar environments, with the hind limbs open and stride length altered]. **Video 4.** [mice circling after infection].

## Data Availability

The datasets supporting the conclusions of this article are included within the article, additional file, and available in the Sequence Read Archive (SRA) of the National Center for Biotechnology Information (https://submit.ncbi.nlm.nih.gov/subs/sra/) under accession NO. PRJNA857467.

## Abbreviations

*N. farcinica*: Nocardia farcinica
PD: Parkinson’s disease
CNS: Central nervous system
hpi: Hours post-infection
PCA: Principal component analysis
KEGG: Kyoto Encyclopedia of Genes and Genomes
DEGs: Differentially expressed genes
TNF: Tumor necrosis factor
MAPK: Mitogen-activated protein kinase
PTS: Phosphotransferase system
LDH: Lactate dehydrogenase
ANGPTL4: Angiopoietin-like 4
MYL5: Myosin light chain 5
BBB: Blood–brain barrier
WISP1: WNT-inducible signaling pathway protein-1
DDIT4: DNA damage-inducible transcript 4
AD: Alzheimer’s disease
ALS: Amyotrophic lateral sclerosis
TH: Tyrosine hydroxylase
LPS: Lipopolysaccharide
ERK: Extracellular regulated protein kinases
JNK: C-jun n-terminal kinase
