# Supplementary material for: Molecular, cellular and neurological consequences of infection by the neglected human pathogen Nocardia
Source: BMC Biol. 2022 Nov 9;20:251. doi: 10.1186/s12915-022-01452-7 (PMC9647956; doi:10.1186/s12915-022-01452-7)
Supplement: Supplementary file 1 — Additional file 1: Figure S1. [Heat map of the Pearson correlation of gene expression between samples for RNA-seq data]. Figure S2. [KEGG pathway analysis of DEGs for N. farcinica and A549 cells]. Figure S3. [KEGG pathway analysis]. Figure S4. [Validation of RNA-seq via qRT–PCR]. Figure S5. [Volcano plot obtained from DESeq2 analysis of DEGs from Nocardia farcinica]. Figure S6. [KEGG pathway analysis of Nocardia at 3 and 6 hpi]. Figure S7. [Analysis of the N. farcinica mutant strains]. Figure S8. [Volcano plot obtained from DESeq2 analysis of DEGs from A549 cells]. Figure S9. [The colony status of the Nocardia strains on the blood plate]. Figure S10. [Analysis of inflammatory factors in serum and brain]. Figure S11. [Original gel images with indicated figures]. [file 12915_2022_1452_MOESM1_ESM.docx]

**Fig. S1**


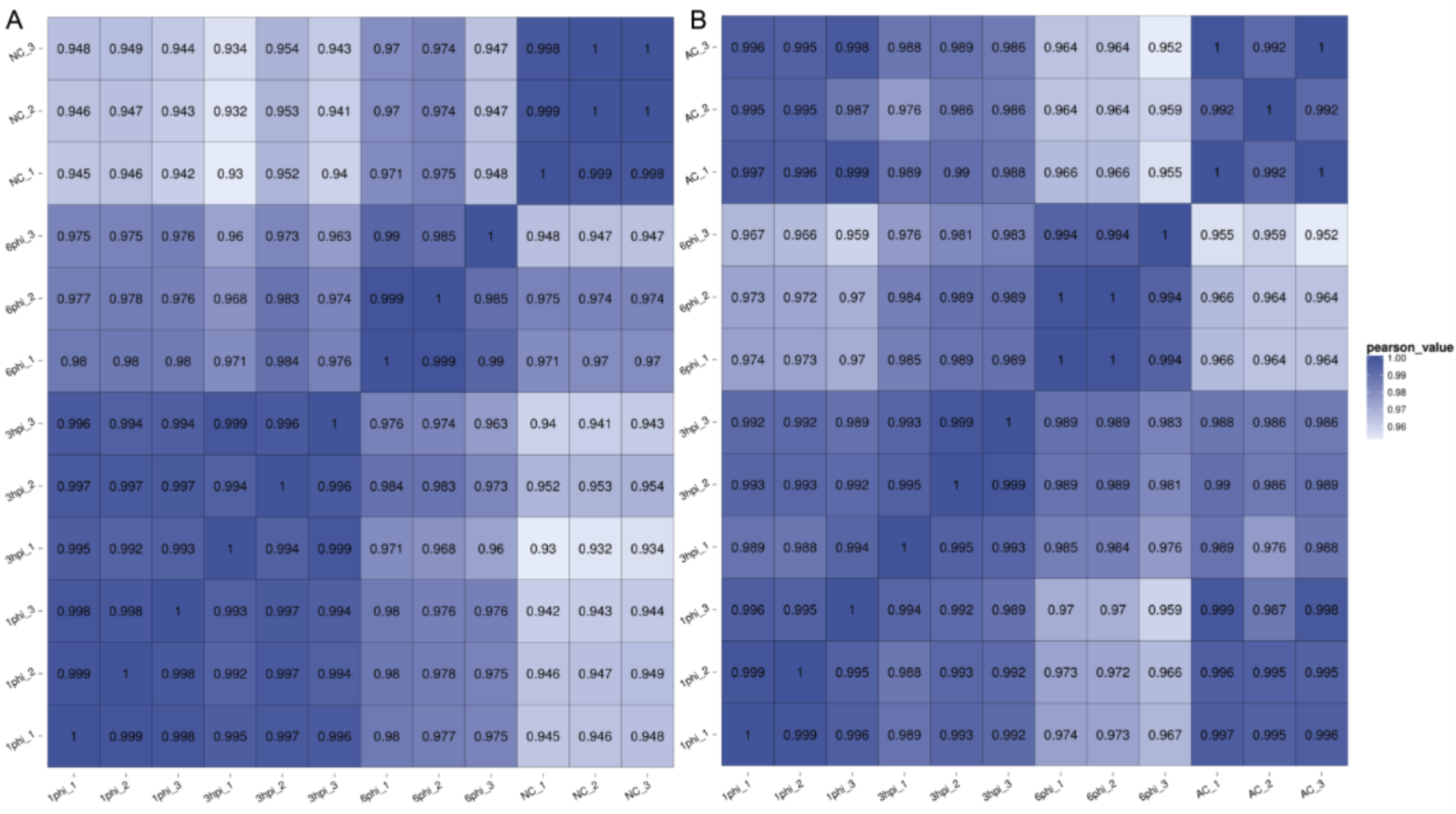


**Fig. S2**

**
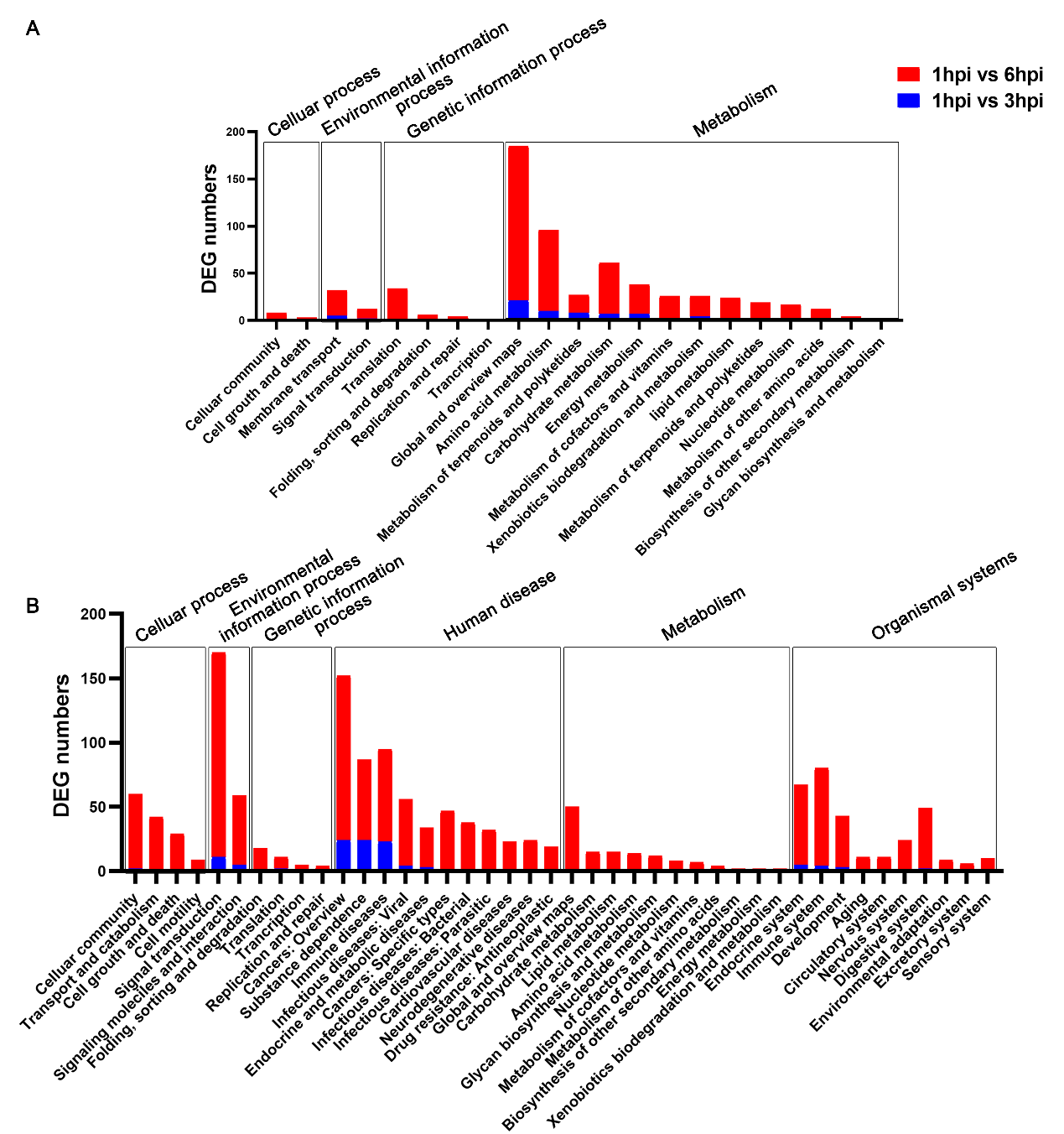
**

**Fig. S3**

**
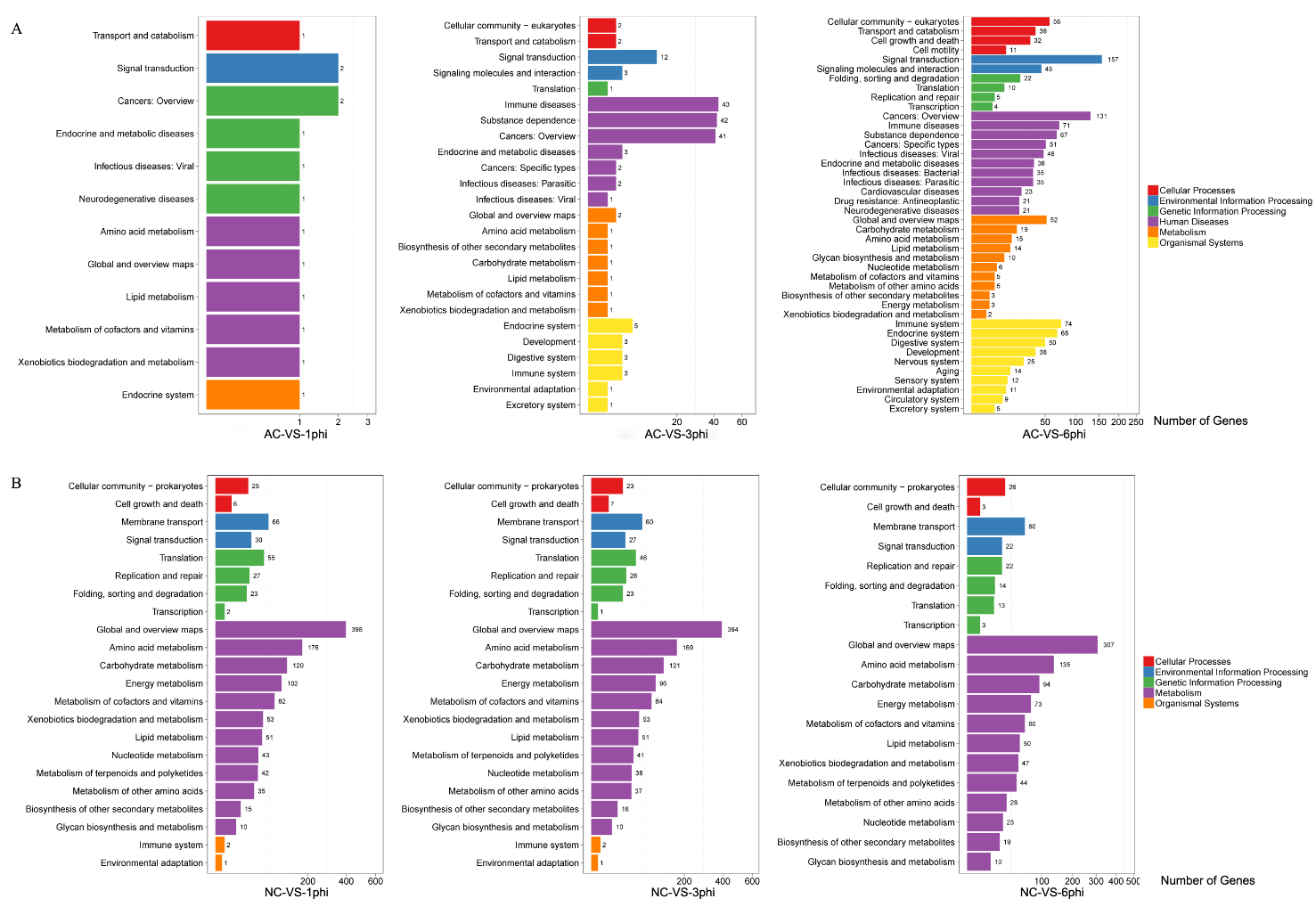
**

**Fig. S4**

**
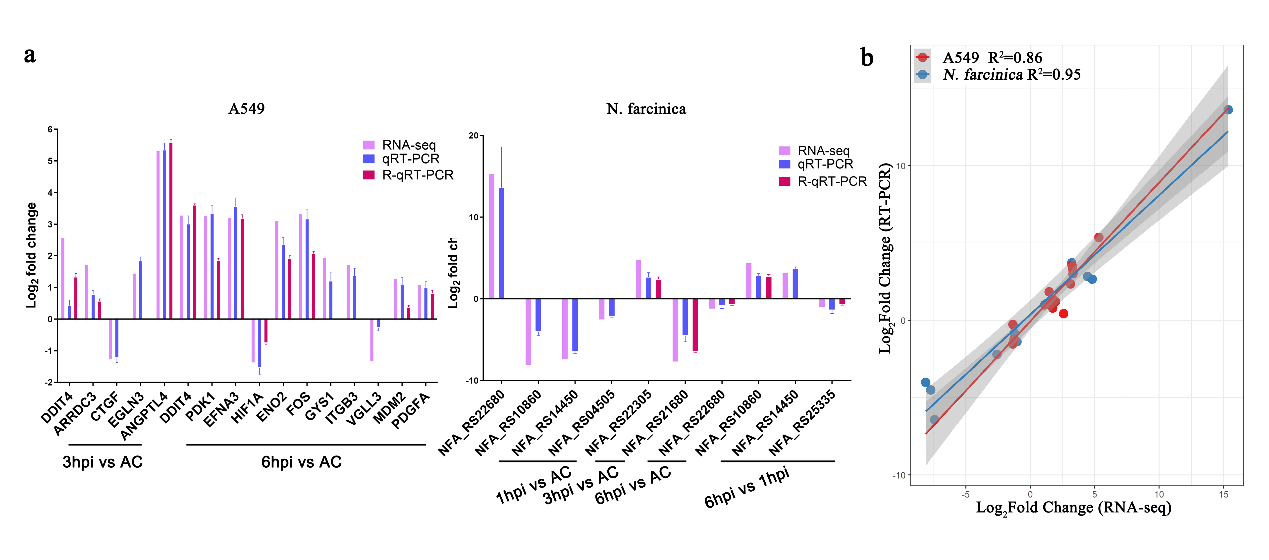
**

**Fig. S5**


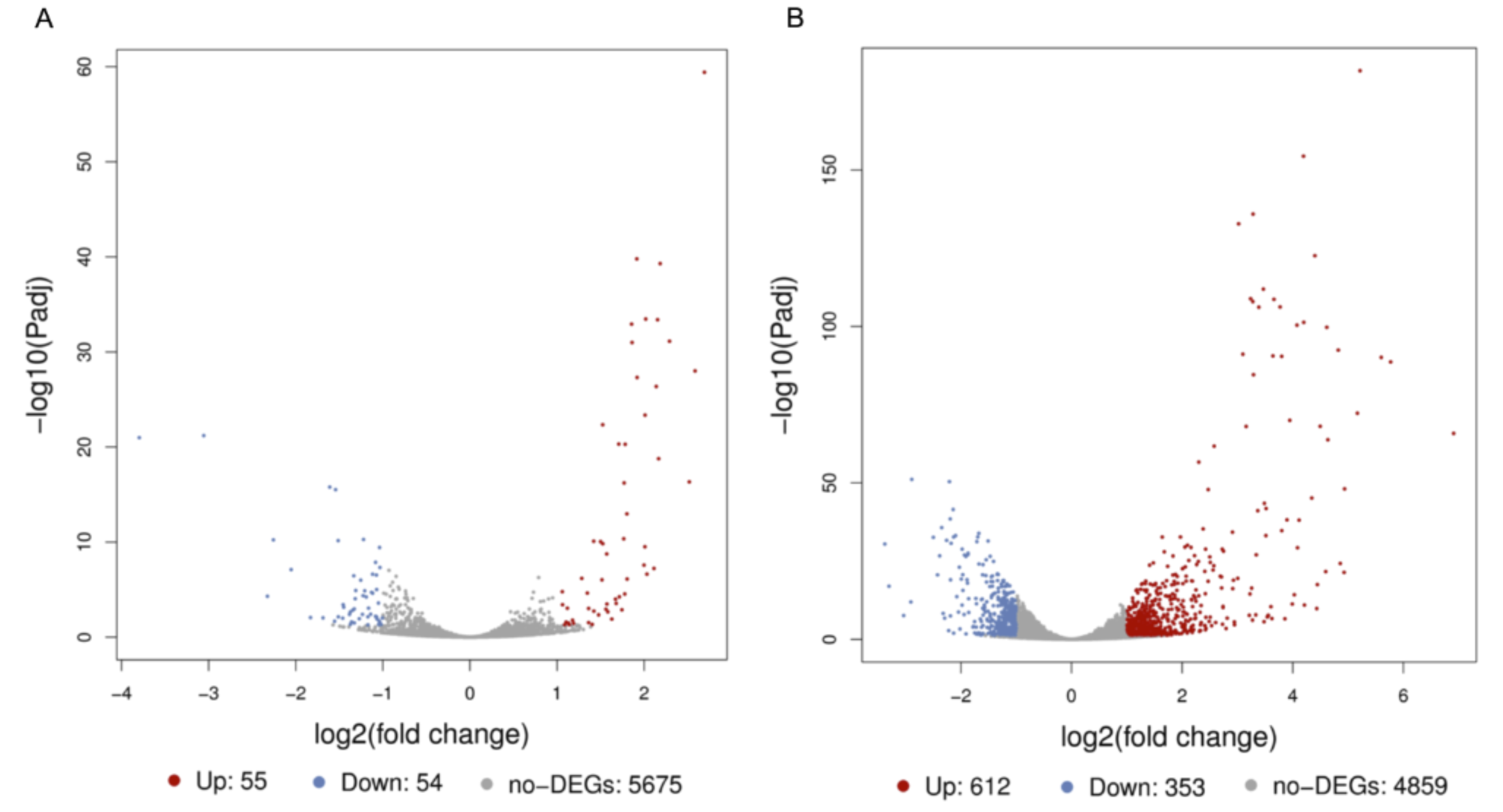


**Fig. S6**


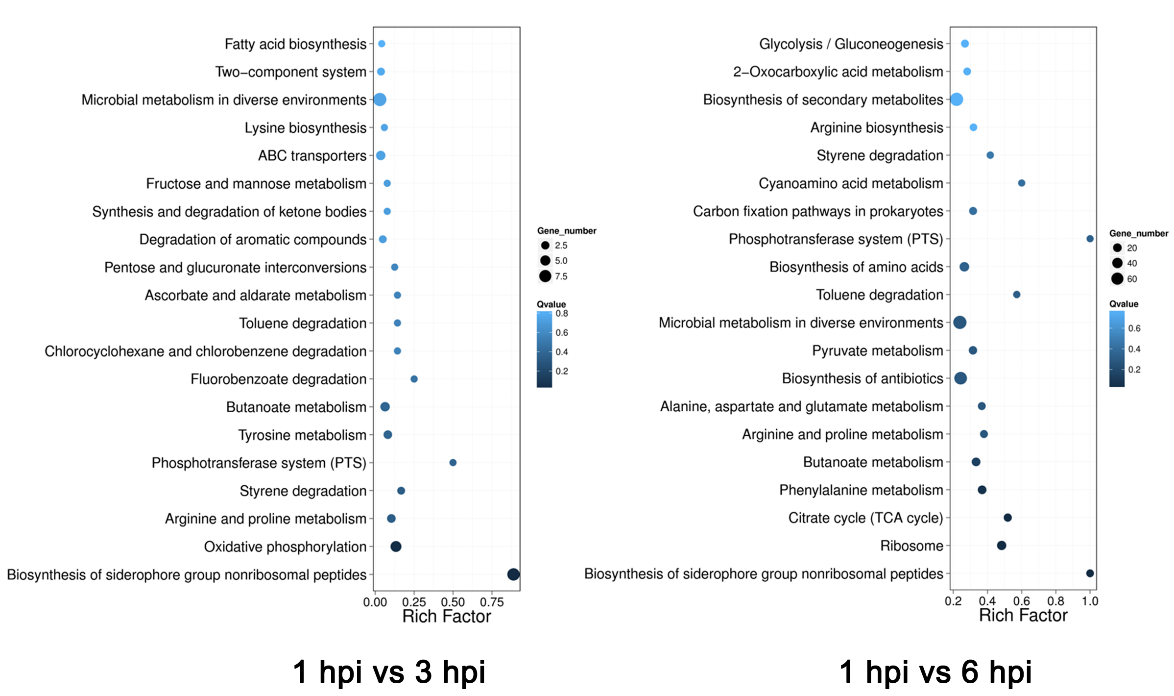


**Fig. S7**

**
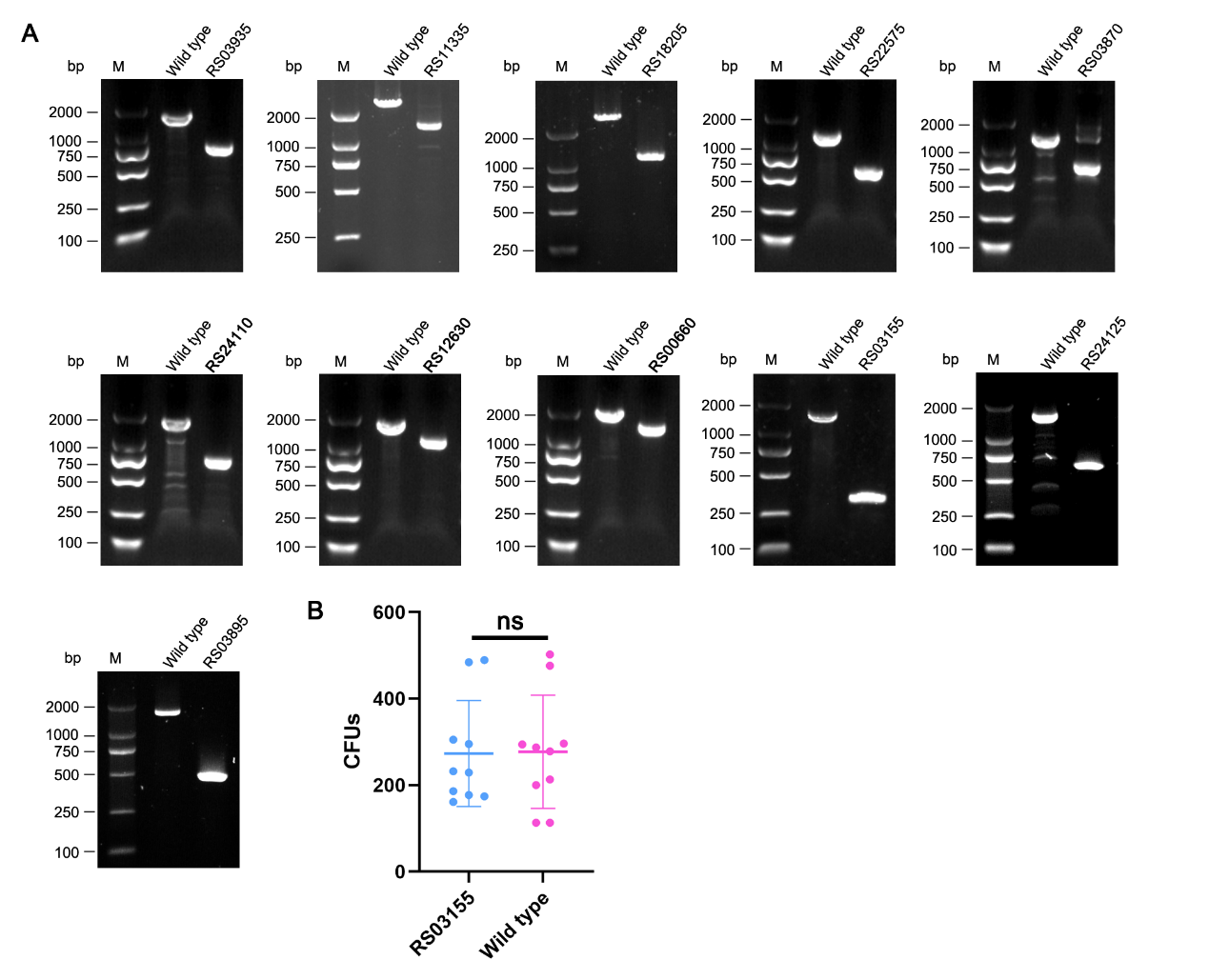
**

**Fig. S8**


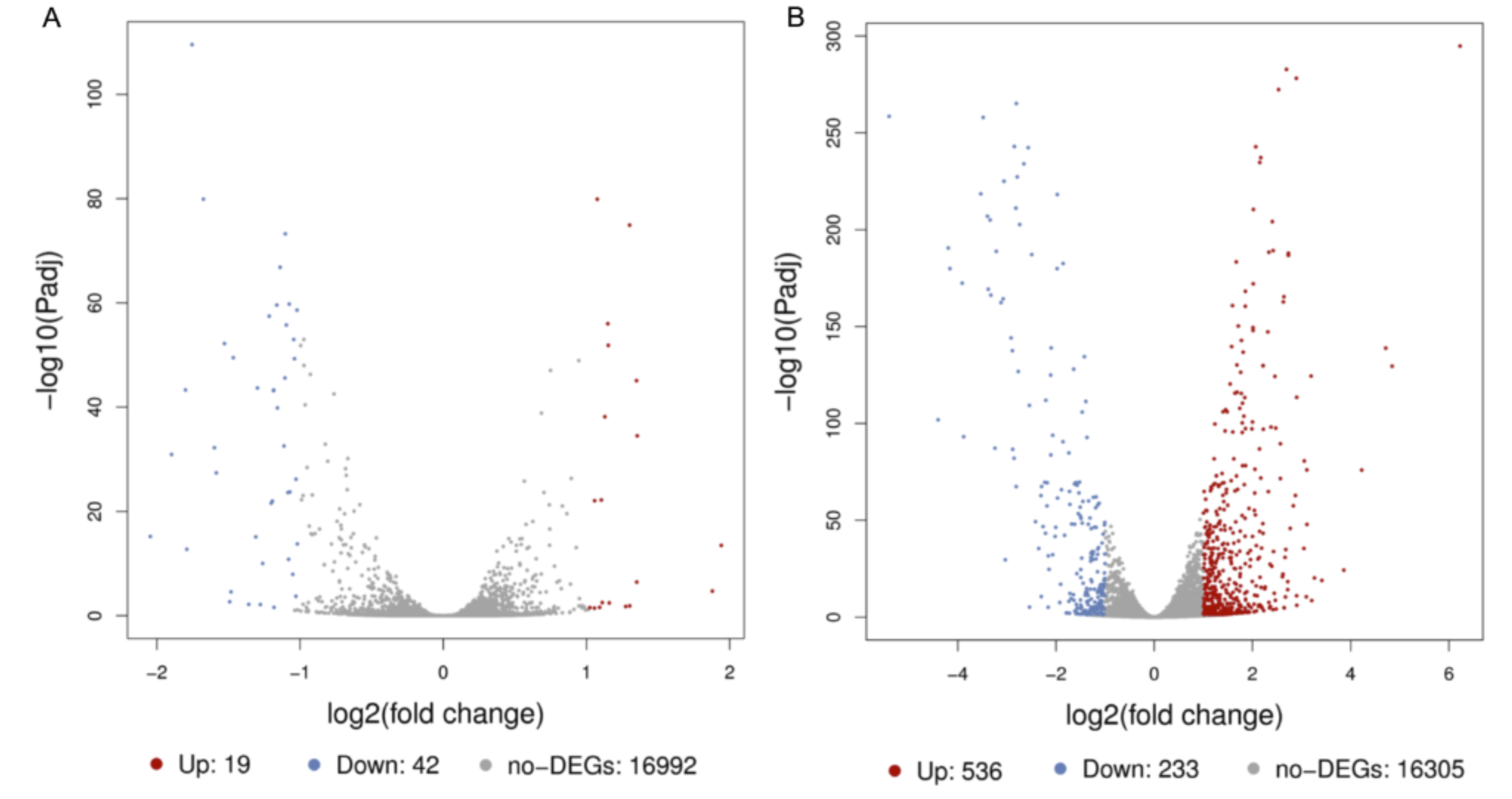


**Fig. S9**


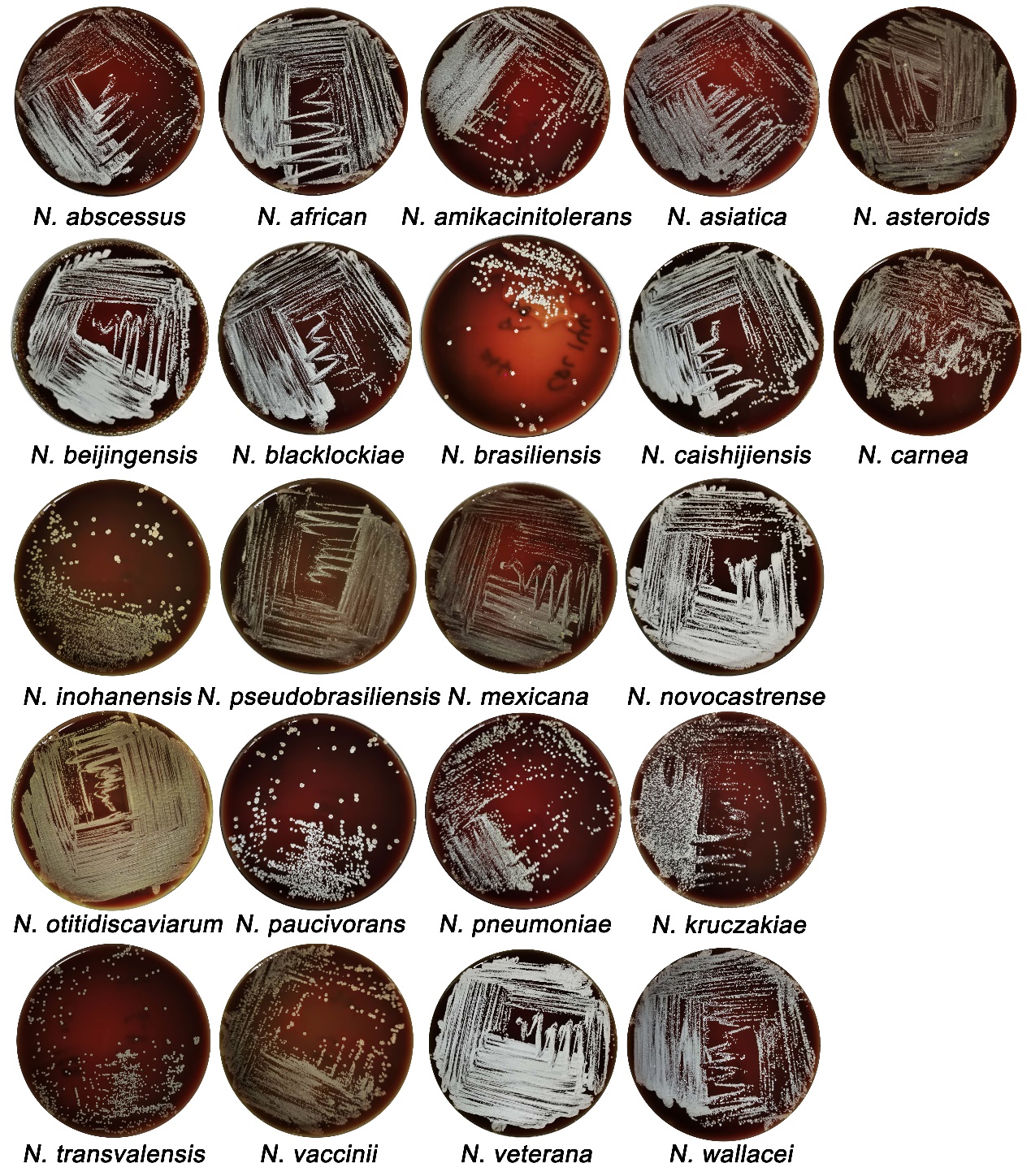


**Fig. S10**


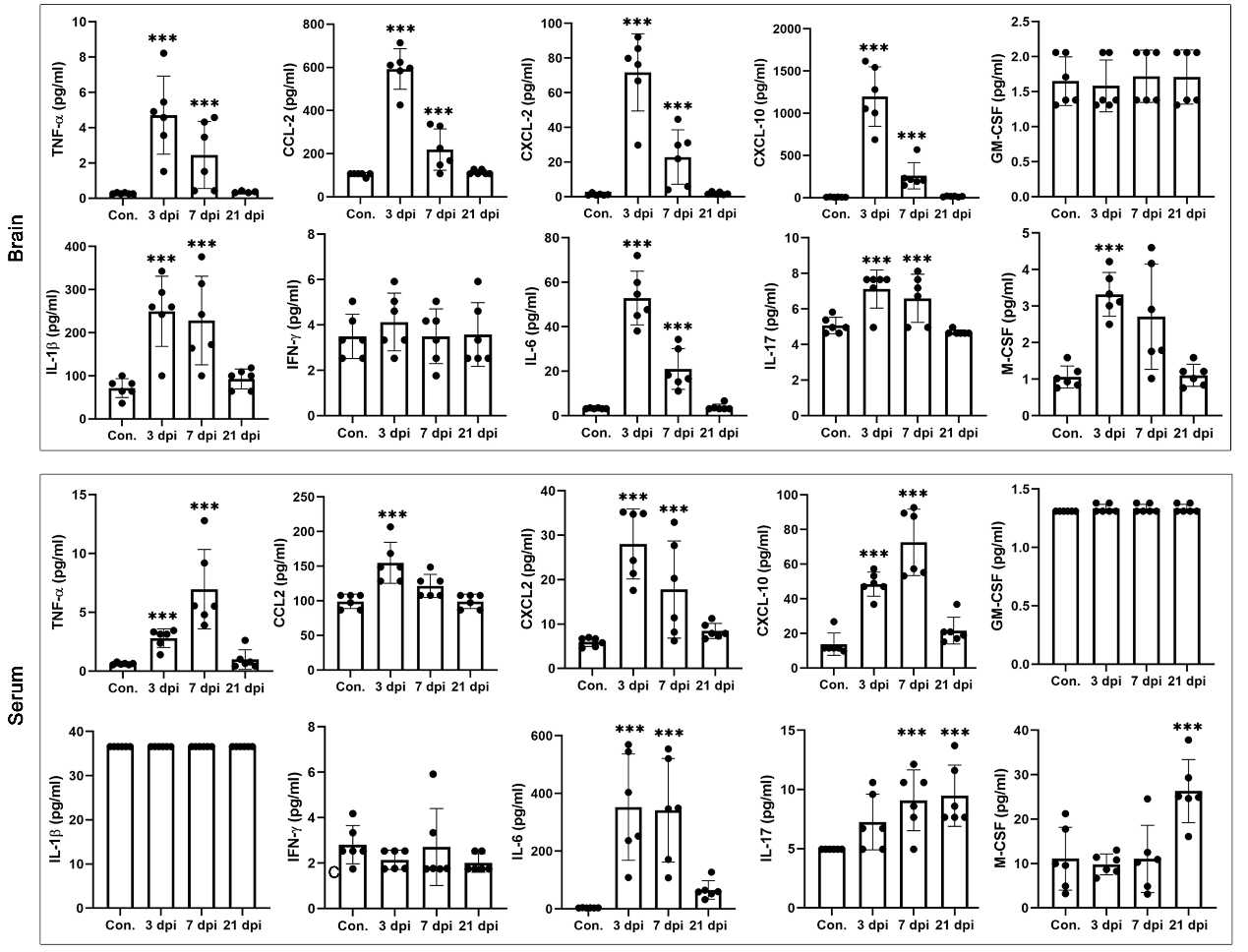


**Fig. S11**


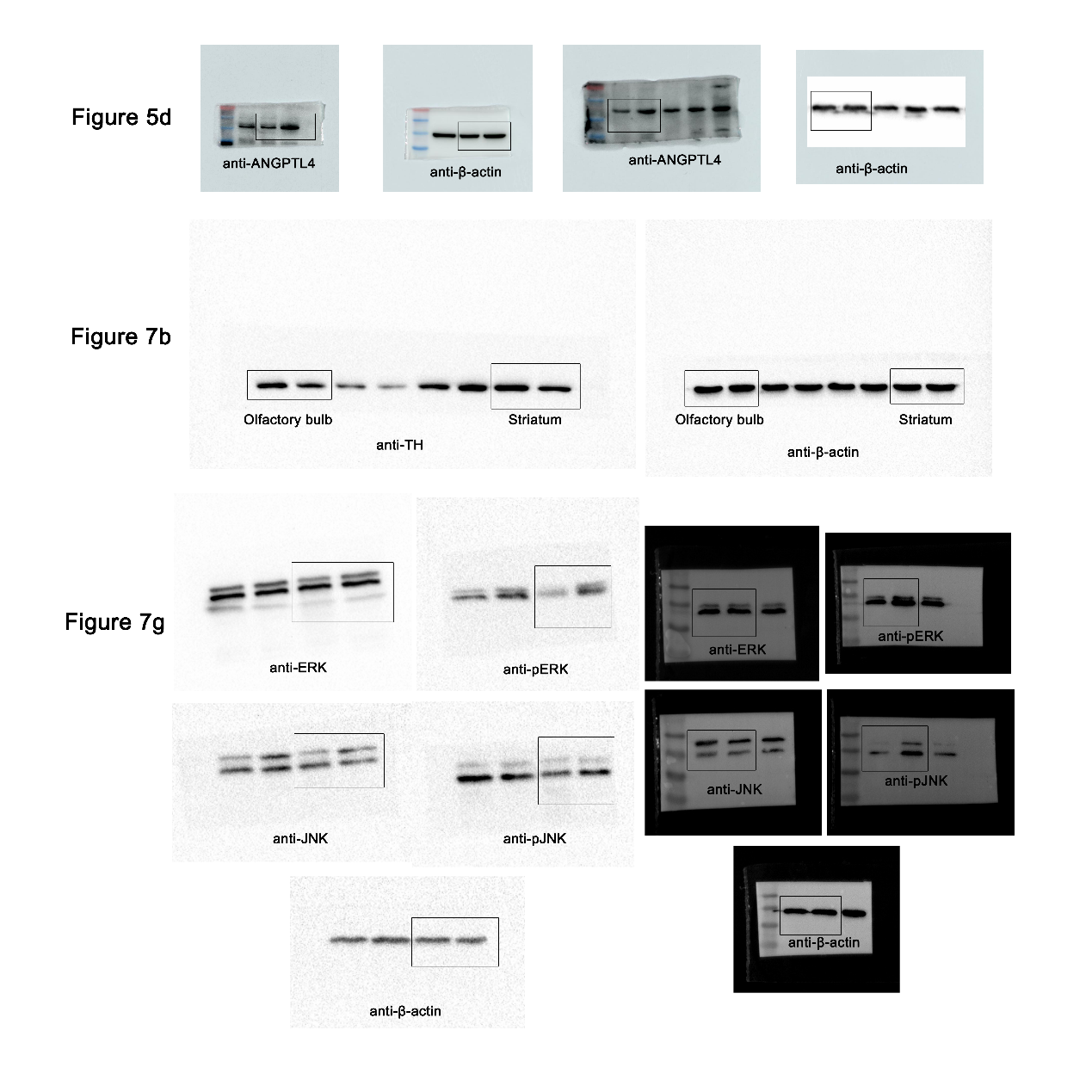


**Additional file 1: Figure legends**

**Fig. S1** Heat map of the Pearson correlation of gene expression between samples for RNA-seq data. A: *Nocardia farcinica*; B: A549 cells.

**Fig. S2** KEGG pathway analysis of DEGs for *N.* *farcinica* (**A**) and A549 cells (**B**).

**Fig. S3** KEGG pathway analysis. A: A549 cells; B: *Nocardia farcinica*

**Fig. S4** Validation of RNA-seq via qRT–PCR. **a** RNA-seq gene expression was verified by qRT–PCR. RNA-seq, the data for dual RNA-seq. qRT–PCR, RNA sample used for sequencing. R-qRT–PCR, RNA sample from the repeated experiment. **b** Correlation analysis of data obtained by RNA-seq and qRT–PCR.

**Fig. S5** Volcano plot obtained from DESeq2 analysis of DEGs from *Nocardia farcinica*. A: The DEGs of 3 hpi compared to 1hpi; B: The DEGs of 6 hpi compared to 1hpi.

**Fig. S6** KEGG pathway analysis of *Nocardia* at 3 hpi and 6 hpi.

**Fig. S7** Analysis of the *N. farcinica* mutant strains. A: The deletion of genes was verified using PCR. The genomes from wild-type and mutant strains were extracted and amplified using primers. The size of the amplified fragment was detected by electrophoresis. B: The bacteria load in BV2 cells 18 h post infection (Student’s two-tailed T-test, ns: *p*>0.05, mean and SD).

**Fig. S8** Volcano plot obtained from DESeq2 analysis of DEGs from A549 cells. A: The DEGs of 3 hpi compared to 1hpi; B: The DEGs of 6 hpi compared to 1hpi.

**Fig. S9** The colony status of the *Nocardia* strains on the blood plate.

**Fig. S10** Analysis of inflammatory factors in serum and brain. The brains of mice were isolated and ground post-infection with *N. farcinica,* and inflammatory factors were analyzed at different times (Student’s two-tailed T-test, ****p*＜0.01, error bars represent SD).

**Fig. S11** Original gel images with indicated figures.

**Table S1** Primers used for qRT-PCR amplification

| Genes | Primer | Sequence (5′ to 3′) |
| --- | --- | --- |
| DDIT4 | F | GGTTTGACCGCTCCACGAG |
|  | R | GGTAAGCCGTGTCTTCCTCC |
| CTGF | F | GGAGTGGGTGTGTGACGAG |
|  | R | CTTCCAGTCGGTAAGCCGC |
| EGLN3 | F | ATCGTTCCCTCTCTGGTTGC |
|  | R | CCATTGCCTCGTGCAGACA |
| ARRDC3 | F | CCGACACCACCTAAATGCGG |
|  | R | TGAGGTAGCGAGTGGTGTCT |
| PDK1 | F | CTCAGGACACCATCCGTTCA |
|  | R | ACCATGTTCTTCTAGGCCTTTCAT |
| ANGPTL4 | F | CCTCTCCGTACCCTTCTCCA |
|  | R | AAACCACCAGCCTCCAGAGA |
| EFNA3 | F | ACAGCCCCATCAAGTTCTCG |
|  | R | GAGTGGGCGTGGAGATGTAG |
| ENO2 | F | TGCACAGGCCAGATCAAGAC |
|  | R | ACAGCACACTGGGATTACGG |
| FOS | F | GGGGCAAGGTGGAACAGTTA |
|  | R | AGTTGGTCTGTCTCCGCTTG |
| GYS1 | F | CGAATGGGGCGACAACTACT |
|  | R | TCTGTGCCAGGAACTTGCAG |
| ITGB3 | F | TTGGAGACACGGTGAGCTTC |
|  | R | GCCCACGGGCTTTATGGTAA |
| MDM2 | F | AGGAGATTTGTTTGGCGTGC |
|  | R | TGAGTCCGATGATTCCTGCTG |
| PDGFA | F | ACTCCTGGAGATAGACTCCGT |
|  | R | GGACAGCTTCCTCGATGCTT |
| HIF1A | F | AAATGAGCTCCCAATGTCGG |
|  | R | CGACGTTCAGAACTTATCCTACCAT |
| VGLL3 | F | TCCAGGGAGACATTGGGTCA |
|  | R | CTGAGCTGTCTCGCCATAGG |
| CACNA1B | F | CCCACAGGGCAGTGGTTC |
|  | R | GTTGGCCGTCTTGTAGGTGA |
| PPP2R5B | F | TAGGTTTTCGAGAAAGCCAGGG |
|  | R | GGGTAGAAAGGAGGATGGCT |
| LRP1 | F | GGACCGCTCTGATGAGTCTG |
|  | R | CAGTCATTGTCATTGTCGCATCT |
| APAF1 | F | CTTCTTCCAGTGTAAGGACAGT |
|  | R | GACCCATCCTGGTTCACCTT |
| DNAH11 | F | TTGGAAAGCGAGGACAACCG |
|  | R | GCATCTCTTGGAATCTCCTGG |
| LOC101928841 | F | TTTCAGGAGGCTTTGAGGGG |
|  | R | CTTGCTGCTCTTCCGGCTTG |
| NFA_RS22680 | F | GACCGCCGAACTGGACACGA |
|  | R | GCCGAAACCGAAGGTCTTGC |
| NFA_RS10860 | F | GGGCTACCGCAAGACCCTCA |
|  | R | GGGTGATCCAGTTGCCCTCC |
| NFA_RS14450 | F | GGACTGGGCCGACGACTACG |
|  | R | CCACAGCAGGCGAACCGA |
| NFA_RS04505 | F | CGCCCAGAGCTTCGACTACC |
|  | R | CGATGGAGGATGCGACCAGAT |
| NFA_RS22305 | F | GCGGCTGCACTTCTACGCG |
|  | R | CGACCTGTTCGAGGGTGGG |
| NFA_RS21680 | F | ATGACCACCGTGGAAACCC |
|  | R | CCCGCAACGACTCGATGTAC |
| NFA_RS22680 | F | GACCGCCGAACTGGACACGA |
|  | R | GCCGAAACCGAAGGTCTTGC |
| NFA_RS10860 | F | GGGCTACCGCAAGACCCTCA |
|  | R | GGGTGATCCAGTTGCCCTCC |
| NFA_RS14450 | F | GGACTGGGCCGACGACTACG |
|  | R | CCACAGCAGGCGAACCGA |
| NFA_RS25335 | F | ACGCCTACACCCTCACCTTCG |
|  | R | TGGTCATCGAGTCCGACAAGG |

**Table S2** Primers used for construction of *N. farcinica* deletion mutants

| Genes | Primer | Sequence (5′ to 3′) |
| --- | --- | --- |
| NFA_RS03935 up | F | CAGAGAATTCCAGGGGACGGCCGATCCAG |
|  | R | AACTCGATGACTGCGCTGGCGTGTGCACCCTCCTTCAT |
| NFA_RS03935 down | F | ATGAAGGAGGGTGCACACGCCAGCGCAGTCATCGAGTT |
|  | R | TACCAAGCTTCACATTGCGCACCACCGGA |
| NFA_RS03935 | F | TTGATCGGGATCGGCACGTTGG |
|  | R | AAGGCGATGCCCAACCATTTCA |
| NFA_RS11335 up | F | ATGAGGATCCAGATCCAGCCCGGCCTCGCCCGC |
|  | R | GTCCATGAGGTTGCCTCTCCTGTCCGGCGAACGCGATCTGTCTTTCCC |
| NFA_RS11335 down | F | AGCCGGGCTCGCGGGAAAGACAGATCGCGTTCGCCGGACAGGAGAGGC |
|  | R | CATGAAGCTTTGGTGGTCTTGCCACAGCCGCTGGG |
| NFA_RS11335 | F | GCTCACGGTGAAGTCGTCCACCAGC |
|  | R | TGGTGAGCCCGGAAACGGTGAATT |
| NFA_RS18205 up | F | AGTCGAATTCATCCTGGCCAGGTAGTGCAGGGCGG |
|  | R | ATGCTGGAGGAACGCTGGGCCGAGTGAGGCCCCAGCCGATGCCGTCCG |
| NFA_RS18205 down | F | GATTTGCGAGCGCGGACGGCATCGGCTGGGGCCTCACTCGGCCCAGCG |
|  | R | ACTGAAGCTTCAGTGTCGACTCGGCGTTCCTGCGC |
| NFA_RS18205 | F | TGACCGACCACCTCCTCCAACGACC |
|  | R | CGAAGCGGTCTCCTACCAGTTCACCA |
| NFA_RS22575 up | F | GACTGGATCCGATGACCGCCTTCGACCAGATG |
|  | R | GGGGCCCACCGATGGTTCATCGGCGGCCTTCCGAG |
| NFA_RS22575 down | F | AGGCCGCCGATGAACCATCGGTGGGCCCCGTCGAA |
|  | R | GATCAAGCTTACTGATAGAGGGGCCAGCCGGC |
| NFA_RS22575 | F | GCGCCAGCCTGCACCTGACCTTCTA |
|  | R | GGCTCCGAAAGCAGATCCCACA |
| NFA_RS03870 up | F | GACTGAATTCAGTTGCCGCCGACCGCGTGT |
|  | R | TCGCCGATCGCGGGGCGCGACTAAGGTTAGCCTAAC |
| NFA_RS03870 down | F | GGCTAACCTTAGTCGCGCCCCGCGATCGGCGAGGCT |
|  | R | GATCAAGCTTAGTGGGCCGAGGAGGTCCGGT |
| NFA_RS03870 | F | GAGCGGCGAAGATCAGGGTGAGGA |
|  | R | CCACCCACGAAAGATTGGATGGAACA |
| NFA_RS24110 up | F | CAGAGAATTCCTGCAACGGTCCGACCGC |
|  | R | AGCGGCGTGCTCCGGTAGAAGGGAATCCCTGG |
| NFA_RS24110 down | F | CCAGGGATTCCCTTCTACCGGAGCACGCCGCT |
|  | R | GATCAAGCTTGGCAGGCGCTCGGACG |
| NFA_RS24110 | F | GGGATACCGAGCGTGCCAAACTG |
|  | R | GGTGTCGTCGTAGAGCGTGATGGTGG |
| NFA_RS12630 up | F | CAGAGAATTCCGCACAGCAGCAC |
|  | R | CCGGCGTCCGCCCCGCTCGGAGCCGACCCGAGGAGGTTCGAT |
| NFA_RS12630 down | F | CCTCCTCGGGTCGGCTCCGAGCGGGGCGGACGCCGGGATTCG |
|  | R | GATCAAGCTTAATGACCGTCACCCGTCC |
| NFA_RS12630 | F | AGGCCCGCGACGAGTGCCAG |
|  | R | TGGTCAGTCCCGCTTGGGCAGGTT |
| NFA_RS00660 up | F | CAGAGAATTCAGGTAGGCCACCGCGTC |
|  | R | CGCATCCTTCCGGAGGTCCGCCGTGTGCCCGGTGCGATGGT |
| NFA_RS00660 down | F | ACCATCGCACCGGGCACACGGCGGACCTCCGGAAGGATGCG |
|  | R | GATCAAGCTTGCCGTCGAGGGCGCCGT |
| NFA_RS00660 | F | ACGCTGACCCCCATCGCGTG |
|  | R | GGTGGGTCCGAAGTAGGCGATACGC |
| NFA_RS03155 up | F | CAGAGGATCCGTCGGTGTCGACTTTCCAG |
|  | R | TGTGGTTTCGCCGGTGTCGTCTCGGGTGTCCGATCCTCCAAAGGTTAAG |
| NFA_RS03155 down | F | AGTTAGGTCAGCTTAACCTTTGGAGGATCGGACACCCGAGACGACACCG |
|  | R | TACCAAGCTTTTCGTCACCGATCCCAGCC |
| NFA_RS03155 | F | CCCGTGCAACACCGAGGTAATGCTAA |
|  | R | ATGCCGAGTCTGGCGGAGGTCAAG |
| NFA_RS24125 up | F | CAGAGGATCCGGCCGTCCTGGTC |
|  | R | CGGGGCGATACGGCGGGGTGATACCCGAGGAGACCACCC |
| NFA_RS24125 down | F | GGGTGGTCTCCTCGGGTATCACCCCGCCGTATCGCCCCG |
|  | R | TACCAAGCTTGCTCCGAGGCCGGG |
| NFA_RS24125 | F | AGCAGTGGTTCGCCAGCGAATTCC |
|  | R | GAACAGCACCAGTGCCAGTCCGCC |
| NFA_RS03895 up | F | GACTGGATCCTCCGACCGTCGATGCGAT |
|  | R | GTCCCGTCGGGGAGCAGGTAAAAAAGAGCTCGGTCGCGTGA |
| NFA_RS03895 down | F | TCACGCGACCGAGCTCTTTTTTACCTGCTCCCCGACGGGAC |
|  | R | ACTGAAGCTTTCAACGCCCCGCACATCCT |
| NFA_RS03895 | F | AGGAACTCCTCGACCACATGACCG |
|  | R | TGTCCTGGTACCTGCCCTCGTCAC |
